# Metabolomics assisted by transcriptomics analysis to reveal metabolic characteristics and potential biomarkers associated with treatment response of neoadjuvant therapy with TCbHP regimen in HER2 + breast cancer

**DOI:** 10.1186/s13058-024-01813-w

**Published:** 2024-04-12

**Authors:** Ningning Zhang, Yuxin Huang, Guanwen Wang, Yimei Xiang, Zhouhong Jing, Junjie Zeng, Feng Yu, Xianjun Pan, Wenqi Zhou, Xiaohua Zeng

**Affiliations:** 1 Department of Breast Cancer Center, Chongqing University Cancer Hospital, Chongqing, China; 2 Department of Breast Cancer Center, School of Medicine, Chongqing University Cancer Hospital, Chongqing University, Chongqing, China; 3 Chongqing Key Laboratory for Intelligent Oncology in Breast Cancer (iCQBC), Chongqing University Cancer Hospital, Chongqing, China

**Keywords:** HER2 + breast cancer, Neoadjuvant therapy, Metabolomics, Transcriptomics, Efficacy, Biomarker

## Abstract

**Background:**

This study aimed to explore potential indicators associated with the neoadjuvant efficacy of TCbHP regimen (taxane, carboplatin, trastuzumab, and pertuzumab) in HER2 + breast cancer (BrCa) patients.

**Methods:**

A total of 120 plasma samples from 40 patients with HER2 + BrCa were prospectively collected at three treatment times of neoadjuvant therapy (NAT) with TCbHP regimen. Serum metabolites were analyzed based on LC-MS and GC-MS data. Random forest was used to establish predictive models based on pre-therapeutic differentially expressed metabolites. Time series analysis was used to obtain potential monitors for treatment response. Transcriptome analysis was performed in nine available pre‑therapeutic specimens of core needle biopsies. Integrated analyses of metabolomics and transcriptomics were also performed in these nine patients. qRT-PCR was used to detect altered genes in trastuzumab-sensitive and trastuzumab-resistant cell lines.

**Results:**

Twenty-one patients achieved pCR, and 19 patients achieved non-pCR. There were significant differences in plasma metabolic profiles before and during treatment. A total of 100 differential metabolites were identified between pCR patients and non-pCR patients at baseline; these metabolites were markedly enriched in 40 metabolic pathways. The area under the curve (AUC) values for discriminating the pCR and non-PCR groups from the NAT of the single potential metabolite [sophorose, N-(2-acetamido) iminodiacetic acid, taurine and 6-hydroxy-2-aminohexanoic acid] or combined panel of these metabolites were greater than 0.910. Eighteen metabolites exhibited potential for monitoring efficacy. Several validated genes might be associated with trastuzumab resistance. Thirty-nine altered pathways were found to be abnormally expressed at both the transcriptional and metabolic levels.

**Conclusion:**

Serum-metabolomics could be used as a powerful tool for exploring informative biomarkers for predicting or monitoring treatment efficacy. Metabolomics integrated with transcriptomics analysis could assist in obtaining new insights into biochemical pathophysiology and might facilitate the development of new treatment targets for insensitive patients.

**Supplementary Information:**

The online version contains supplementary material available at 10.1186/s13058-024-01813-w.

## Introduction

Breast cancer (BrCa) has replaced lung cancer as the most commonly diagnosed cancer in women worldwide [1], and is the leading cause of cancer-related mortality in females [2]. Approximately 15–20% of all instances of BrCa are human epidermal growth factor receptor 2 positive (HER2+) [3], which exhibits aggressive biological and clinical behavior and is linked to disease recurrence, metastasis, and unfavorable prognoses [4, 5]. However, in the last two decades, there have been many advances in HER2-targeted drugs, such as the monoclonal antibodies trastuzumab (H) and pertuzumab (P); tyrosine kinase inhibitors lapatinib, neratinib, tucatinib and pyrotinib; and the antibody-drug conjugates T-DM1 and trastuzumab deruxtecan (T-DXd), which have dramatically improved the outcomes of HER2 + BrCa patients [6].

Neoadjuvant therapy (NAT) can reduce the size of locally advanced BrCa tumors, decrease the subclinical micro-metastatic illness, and improve the rate of breast-conserving surgeries [7–9]. Meanwhile, NAT provides a unique chance to evaluate the response of patients with BrCa to different treatments [10]. Currently, the pathologic complete response (pCR) is a commonly used assessment tool for evaluating the response to NAT and is one of the most important target endpoints of NAT due to its correlation with favorable patient outcomes [11]. For patients with high-risk characteristics who are eligible for NAT, dual HER2-blockade with trastuzumab and pertuzumab coupled with chemotherapy is advised as the gold standard of care worldwide, pending medication availability [12]. In addition, in clinical practice, the regimen of taxane, carboplatin, and trastuzumab in combination with pertuzumab (TCbHP) has become the preferred and the most commonly used neoadjuvant regimens for HER2 + BrCa in China [13]. However, clinical challenges might arise when cancer cells are resistant to currently available HER2 inhibitors. Currently, not all HER2 + BrCa patients can benefit from NAT, and approximately 40-50% of patients cannot achieve pCR at surgery, even if the HP based regimen is used [14–16]. At present, there are no effective indicators for predicting pathological response to NAT in the clinic. Therefore, exploring biomarkers to assess the pathogenic response to NAT is crucial and will assist in the exploration of novel strategies to overcome resistance and have significant impact on the customized treatment of HER2-positive BrCa.

A proposed mechanism of HER2 + BrCa cell resistance to anti-HER2 therapy is altered metabolism [17], a well-known hallmark of malignancy [18]. In fact, HER2-mediated signaling has been linked to the activation of certain metabolic pathways, underscoring the importance of metabolic dysregulation in sustaining unregulated growth, proliferation, and treatment resistance in HER2 + BrCa cells [19]. Panels of metabolites have been used as biomarkers for early diagnosis, grading, staging, molecular typing discrimination, and prognosis prediction of BrCa [20–27]. The changes in metabolite profiles might be caused by the complex interactions between multiple environmental factors and gene expression [18]. Transcriptomics could assist in identifying certain molecular responses and generating theories on the underlying processes involved [28]. The integration of transcriptomics and metabolomics may provide more information on tumor pathophysiology than any technique used separately [29]. However, research identifying biomarkers for evaluating the therapeutic response of HER2 + BrCa patients to NAT via metabolomics and/or transcriptomics is still sparse. Furthermore, the existing studies are most focused on single-targeted therapy rather than dual-targeted therapy.

In this study, liquid chromatography-mass spectrometry (LC-MS) and gas chromatography-mass spectrometry (GC-MS) platform-based untargeted metabolomics were used to determine the metabolites in 120 plasma samples from 40 patients with HER2 + BrCa who received NAT via the TCbHP regimen at three different time points (pre-treatment, under treatment, post-treatment). Moreover, transcriptome analysis of nine available biopsy samples was performed to identify differentially expressed genes (DEGs) between non-PCR patients and pCR patients. Pathways significantly altered in association with drug resistance were identified by integrating metabolic and transcriptomic data. Quantitative real-time polymerase chain reaction (qRT-PCR) was used to verify several DEGs in trastuzumab-resistant and trastuzumab-sensitive cell lines. This research was conducted to explore both possible biomarkers and pathways associated with therapeutic response and to gain insight into the dynamic changes in efficacy-related metabolites at the different times of dual-targeted NAT with TCbHP. Moreover, a prediction model of the treatment response to TCbHP in patients with HER2 + BrCa was established based on differentially expressed metabolites (DEMs) using random forest (RF), which may help to identify insensitive patients before treatment.

## Methods

### Patient population

HER2-amplified BrCa patients who underwent and successfully completed NAT with the TCbHP regimen and subsequent surgery at the Department of Breast Cancer Center, Chongqing University Cancer Hospital, from July 20, 2020, to May 28, 2021, were included in this prospective analysis. The study received approval from the Chongqing University Cancer Hospital’s ethics committee (CZLS2022022-A) and was carried out in strict conformity with the Good Clinical Practice guidelines and the Declaration of Helsinki. Informed consent form was signed by every patient.

The detailed other inclusion criteria were as follows: (1) histologically diagnosed with invasive breast carcinoma by core needle biopsy and subjected to IHC analysis with paraffin-embedded tumor samples biopsied before NAT. At least 1% immunoreactivity for either the estrogen receptor (ER) or progesterone receptor (PR) in tumor cell nuclei was required for a positive result. According to the 2018 American Society of Clinical Oncology (ASCO)/College of American Pathologists (CAP) Clinical Practice Guidelines, HER2 overexpression was indicated by a score of 3 + immunoreaction intensity or 2 + immunoreaction intensity with HER2 amplification by fluorescence in situ hybridization (FISH) [30]; (2) the lack of metastases, as determined by ultrasound, magnetic resonance imaging (MRI), computed tomography (CT), bone scan, and/or positron emission tomography (PET)/CT; When metastases to the axillary lymph nodes was either suspected or discovered, fine needle aspiration cytology was carried out; (3) having healthy kidney, liver, and hematopoietic systems as well as an echocardiography without significant cardiac arrhythmia or heart failure; (4) received the TCbHP regimen, which included carboplatin [area under curve (AUC) = 6], docetaxel [75 mg/m^2^, without dose escalation], a loading dose of trastuzumab (8 mg/kg) with a maintenance dose of 6 mg/kg, and a loading dose of pertuzumab (840 mg) with a maintenance dose of 420 mg every three weeks for six cycles.

The following conditions were excluded from the study: a history of prior malignancy (apart from inactive non-melanoma skin cancer and in situ cervical carcinoma), complicated with metabolic disorder syndrome, a current infection, and other concomitant illnesses that might impact medication tolerance or hamper compliance.

### Evaluation of the NAT pathological response

Pathological response was gauged using the semi-quantitative Miller-Payne (MP) grading method. This gauges the percentage reduction in invasive tumor volume and cellularity following NAT based on the pathological evaluation of surgical samples [31]. In our investigation, pCR was determined by experienced pathologists to be the absence of residual invasive disease after surgery in both the breast (MP grade 5) and axillary lymph nodes (ypT0/is ypN0).

### Sample collection

A total of 120 blood specimens from 40 patients were collected at three time points of NAT (baseline, T1; after 2 cycles, T2; after 6 cycles, before surgery, T3). Blood samples were taken from the elbow vein in the fasting state in the morning, kept in ethylenediaminetetraacetic acid vacuum tubes (BD Vacutainer, Franklin Lakes, NJ, USA), and centrifuged for 10 min at 3000 rpm at 4 °C. Immediately after separation, the serum was kept at -80 °C for further examination. A total of 1–2 cores of biopsies were taken from the breast tumor at the diagnosis and placed promptly frozen in liquid nitrogen before being stored at -80 °C until use.

### Non-targeted metabolomic analysis

#### GC-MS detection

The samples stored at -80 ℃ were thawed at room temperature. The following steps were taken to prepare the samples for GC-MS analysis: briefly, 450 µL methanol and acetonitrile (2/1, vol/vol) were used to extract the metabolites from the cecal content sample (150 µL). 50 µL BSTFA (with 1% TMCS) and 20 µL n-hexane were used to oxidate and derive the metabolites. During sample processing, ten different internal standards (C8/C9/C10/C12/C14/C16/C18/C20/C22/C24) were applied. Before the GC-MS analysis, the samples were left at room temperature for 30 min. Equal aliquots from each sample were combined to create the quality control (QC) sample.

The derivatized samples were examined using an Agilent 5977 A MSD system and an Agilent 7890B gas chromatography system (Agilent Technologies Inc., CA, USA). The derivatives were separated using a DB-5MS fused-silica capillary column (30 m × 0.25 mm × 0.25 μm, Agilent J&W Scientific, Folsom, CA, USA). As the carrier gas, helium (> 99.999%) was pumped through the column at a constant flow rate of 1 mL/min. The initial oven temperature was 60℃, held at 60 °C for 0.5 min, ramped to 125℃ at a rate of 8℃/min, to 210℃ at a rate of 5℃/min, to 270℃ at a rate of 10℃/min, to 305℃ at a rate of 20℃/min, and finally held at 305℃ for 5 min. The temperature of MS quadrupole and ion source (electron impact) was set to 150 and 230℃, respectively. The collision energy was 70 eV. Mass spectrometric data was acquired in a full-scan mode (m/z 50–500), and the solvent delay time was set to 5 min.

The QC sample was created by combining aliquots from each sample. Throughout the analytical run, the QCs were injected at regular intervals (every 10 samples) to generate a set of data from which repeatability could be evaluated.

#### LC-MS detection

At normal temperature, the samples frozen at -80 °C were defrosted. The following sample preparation procedures were used for LC-MS analysis: in brief, 150 µL of sample and 10 µL of L-2-chlorophenylalanine (0.06 mg/mL) dissolved in methanol were added to a 1.5 mL Eppendorf tube, and the tube was vortexed for 10 s. Then, 450 µL of an ice-cold mixture of acetonitrile and methanol (2/1, vol/vol) was added. The solutions were vortexed for 1 min, and the entire batch of samples was extracted using an ultrasonic device for 10 min in an ice-water bath before being kept at -20 °C for two hours. The extract was centrifuged for 10 min at 4 °C (13,000 rpm). A freezing concentration centrifugal drier was used to dry 150 µL of the supernatant in a glass vial. With the use of crystal syringes and 0.22 μm microfilters, the supernatants (150 µL) from each tube were collected and subsequently transferred to LC vials. Prior to LC-MS analysis, the vials were kept at -80 °C. A pooled sample made from an aliquot of each sample was used to create the QC samples.

To examine the metabolic profile in both ESI positive and ESI negative ion modes, a Dionex Ultimate 3000 RS UHPLC equipped with a heated electrospray ionization (ESI) source and a Q Exactive plus quadrupole-Orbitrap mass spectrometer (Thermo Fisher Scientific, Waltham, MA, USA) was used. In both positive and negative modes, an ACQUITY UPLC HSS T3 column (1.8 μm, 2.1 × 100 mm) (Waters Corporation Milford, Milford, MA, USA) was used. The binary gradient elution system consisted of (A) water (containing 0.1% formic acid, v/v) and (B) acetonitrile (containing 0.1% formic acid, v/v) and separation was achieved using the following gradient: 0.01 min, 5% B; 2 min, 5% B; 4 min, 30% B; 8 min, 50% B; 10 min, 80% B; 14 min, 100% B; 15 min, 100% B; 15.1 min, 5% and 16 min, 5%B. The flow rate was 0.35 mL/min and column temperature was 45℃. All the samples were kept at 10℃ during the analysis. The injection volume was 2 µL.

The mass range was from m/z 100 to 1,000. The resolution was set at 70,000 for the full MS scans and 17,500 for HCD MS/MS scans. The Collision energy was set at 10, 20 and 40 eV. The mass spectrometer operated as follows: spray voltage, 3800 V (+) and 3200 V (−); sheath gas flow rate, 35 arbitrary units; auxiliary gas flow rate, 8 arbitrary units; capillary temperature, 320 °C; Aux gas heater temperature, 350 °C; S-lens RF level, 50.

#### The metabolomic data processing

The plasma samples from patients in non-pCR (*N* = 19) and pCR groups (*N* = 21) at T1, T2, and T3 time points were termed as the A, B, C and D, E, F groups, respectively. The metabolomic analyses were based on these patients and groups.

To enable rapid data retrieval, the collected GC-MS raw data in.D format were converted to ABF format using Analysis Base File Converter program. The data were subsequently entered into the program MS-DIAL, which carries out peak detection, peak identification, characterization, MS2Dec deconvolution, peak alignment, peak filtering, and missing value interpolation. The LUG database (Untargeted metabolites database of GC-MS from Lumingbio) was used for the annotation of metabolites. A data matrix was derived. The sample information, the name of each substance’s peak, retention duration, retention index, mass-to-charge ratio, and signal intensity were included in the three-dimensional matrix. After screening, all peak signal intensities in each sample were segmented and normalized according to internal standards with relative standard deviation (RSD) greater than 0.3. Redundancy elimination and peak merging were carried out after the data had been standardized to produce the data matrix.

Progenesis QI V2.3 (Nonlinear, Dynamics, Newcastle, UK) was used to process the original LC-MS data for baseline filtering, peak identification, integral, retention time correction, peak alignment, and normalization. The main parameters used were 5% production threshold, 10 ppm product tolerance, and 5 ppm precursor tolerance. Using the Human Metabolome Database (HMDB) (https://hmdb.ca/), LIPID MAPS (V2.3) (https://lipidmaps.org/), Metlin (https://metlin.scripps.edu), EMDB, PMDB, and self-built databases to conduct qualitative analysis, compounds were identified based on the precise mass-to-charge ratio (M/z), secondary fragments, and isotopic distribution. The retrieved data were then subjected to additional processing, including removal of any peaks with missing values (ion intensity = 0) in more than 50% of the groups, replacement of zero values with half of the minimum values, and screening in accordance with the qualitative outcomes of the compound. Additionally, compounds that produced results of less than 36 (out of 60) points were declared incorrect and eliminated. The data from the positive and negative ions were integrated into a data matrix.

Then the matrix was imported into R (R package MixOmics) to perform unsupervised analysis of principal component analysis (PCA) to observe the general distribution among the samples and the stability of the entire analytic process. The metabolites that differed across groups were identified using supervised analysis of orthogonal partial least-squares discriminate analysis (OPLS-DA) (R package MetaboAnalystR), which were offen used to maximize the global metabolic variations among groups. Seven folds cross-validation and 200 response permutation testing (RPT) were employed to assess the model’s quality and minimize overfitting. The total contribution of each variable to group discrimination was ranked using the variable importance of projection (VIP) values derived from the OPLS-DA model. DEMs with VIP values higher than 1.0 and p-values lower than 0.05 were chosen. Hierarchical clustering analysis (pheatmap, R package pheatmap) based on these DEMs were utilized to demonstrate the expression pattern of DEMs in different groups and samples. The enriched pathway analysis of changed metabolites was performed using Kyoto Encyclopedia of Genes and Genomes (KEGG) database (http://www.genome.jp/KEGG/pathway.html) with the Hypergeometric Test to calculate significantly perturbed pathways. The pathway impact is the sum of the importance of the matched metabolites normalized by the sum of the importance of all the metabolites in each metabolic pathway.

### Transcriptomic analysis

RNA isolation and high-throughput RNA sequencing (RNA-Seq) were performed by Oebiotech Corp (Shanghai, China). The Spin Column Bacterial Total RNA Purification Kit (Sangon Biotech, Shanghai, China) was used to extract the total RNA. RNA purity and quantification were evaluated using the Agilent 2100 bioanalyzer (Agilent Technologies, Santa Clara, CA, USA). The NanoDrop2000 spectrophotometer (Thermo Fisher Scientific, Waltham, MA, United States) was used to calculate the concentration. Integrity number (RIN) ≥ 7, 28 S/18S ≥ 0.7, and total RNA concentration greater than 0.5 µg were the selection criteria for RNA samples. For some of the enrolled patients had pathologic confirmation of BrCa when they consulted at our hospital or had insufficient residual tissue for transcriptomics test after pathology diagnosis, only 21 pre-treatment puncture specimens from these 40 patients were available for RNA extraction. After the quality control, only nine samples (43%), including six samples from patients in the non-pCR group (a03, a06, a12, a15, a17, a19) and three samples from patients in pCR group (d02, d08, d10), met the experimental requirements for RNA-Seq libraries construction; the other 11 samples had the RIN < 6–28 S/18S < 0.7, and one sample did not have sufficient total RNA for further experimentation. The libraries were constructed using VAHTS Universal V6 RNA-seq Library Prep Kit according to the manufacturer’s instructions. The transcriptome sequencing and analysis were conducted by OE Biotech Co., Ltd. (Shanghai, China).

The libraries were sequenced on a llumina Novaseq 6000 platform and 150 bp paired-end reads were generated. About 48.13 M raw reads for each sample were generated. Raw reads of fastq format were firstly processed using fastp and the low quality reads were removed to obtain the clean reads. Then about 6.7G CleanData for each sample were retained for subsequent analyses. The clean reads were mapped to the human reference genome GRCh38.p13 using HISAT2. Fragments per kilobase million (FPKM) of each gene was calculated and the read counts of each gene were obtained by HTSeq-count. Covariance-based PCA analysis were performed on the top 2000 highly variable genes with the highest degree of variation using R (v 3.2.0) to evaluate the biological duplication of samples.

Differential expression analysis was performed using the DESeq2 with negative binomial distribution (NB) test. Q value < 0.05 and foldchange ≥ 2 or foldchange ≤ 0.5 was set as the threshold for significantly DEGs. Hierarchical cluster analysis of DEGs was performed using R (v 3.2.0) to demonstrate the expression pattern of genes in different groups and samples.

Based on the hypergeometric distribution, Gene Ontology (GO, which provide annotation information of biological process, molecular function, and cellular component, http://www.Geneontology.org/) and KEGG pathway enrichment analysis of DEGs were performed to screen the significant enriched term using R (v 3.2.0), respectively.

### Integrative analysis of metabolome and transcriptome

In this study, integrative analyses of gene expression differences (derived from nine patients including six patients from non-pCR group and three patients from pCR group) and metabolism differences (derived from 40 patients including 19 patients from non-pCR group and 21 patients from pCR group) between the non-pCR group and the pCR group at baseline were explored by transcriptomic analysis and untargeted metabolomic analysis. Based on the top 20 DEGs and DEMs, Spearman correlation coefficients were calculated by R and the cluster analysis heatmap was drawn. Then, all DEGs and DEMs were mapped to the KEGG pathway database, and common pathways information of them was acquired.

### Cell lines and quantitative reverse transcription-polymerase chain reaction (qRT-PCR)

HER2 + BrCa cell lines, including trastuzumab-sensitive SK-BR-3 and BT-474 cells, primary trastuzumab-resistant JIMT-1 cells, and acquired trastuzumab-resistant SK-BR-3-HR cells (generated in our laboratory from the parental cell line SK-BR-3 by exposing the cells to gradually increasing concentrations of trastuzumab for 6 months), were cultured in appropriate medium. TRIzol solution (Thermo Fisher’s) was used to extract the total RNA from these cell lines, and a PrimeScript RT reagent kit (Yeasen, China) was used to reverse-transcribe the RNA into cDNA. qRT-PCR was performed using a LightCycler 480 (Roche) with a SYBR-based detection system, and specific primers were used to measure the relative mRNA expression levels of all the genes. The relative levels of the target genes to the control β-actin mRNA transcripts in each sample were analyzed by the 2-^ΔCt^ method. Each experiment was run in triplicate. The primers utilized and their sequences are listed in the Supplementary Table 1.

### Statistical analysis

Wilcoxon test was used for continuous variable (age at diagnosis), Chi-square test or Fisher exact test were used to compare dichotomous variables in other clinicopathological features analyses. The significance of metabolites in two groups or multiple groups was calculated by Wilcoxon test and Kruskal-Wallis test, respectively. The metabolites with VIP > 1.0 and *P* < 0.05 between two groups were considered as DEMs. RF was performed to screen potential metabolites for the prediction of treatment response to NAT (randomforest package) based on the pre-therapeutic DEMs between non-pCR patients and pCR patients. The two important parameters used in the RF classifier are ntree (number of trees) and mtry (the number of features to choose the best subset). The ntree parameter was set to 500 trees. The mtry was set to the default value (sqrt(p) where p is number of variables in the metabolomic data). The area under receiver operating characteristic (ROC) curve (AUC) (R package pROC) was applied to evaluate the performance of the predictive model based on the four pre-therapeutic metabolites selected by RF analysis. The correlation of the four pre-therapeutic metabolites with MP grade, and the correlation of DEMs and DEGs were calculated by Spearman correlation coefficients. The trends analyses of DEMs expression between non-pCR and pCR patients were performed by Mfuzz R package. The qRT-pCR results of several DEGs were presented as mean ± SD, and Student t test was used to generate *P* value between two cell lines. The visualization of results was performed by R (v 3.2.0) and GraphPad Prism software (v8.0). Unless otherwise stated, a variable was deemed to be statistically significant at *P* < 0.05.

## Results

### Patient clinical characteristics

**Fig. 1:**
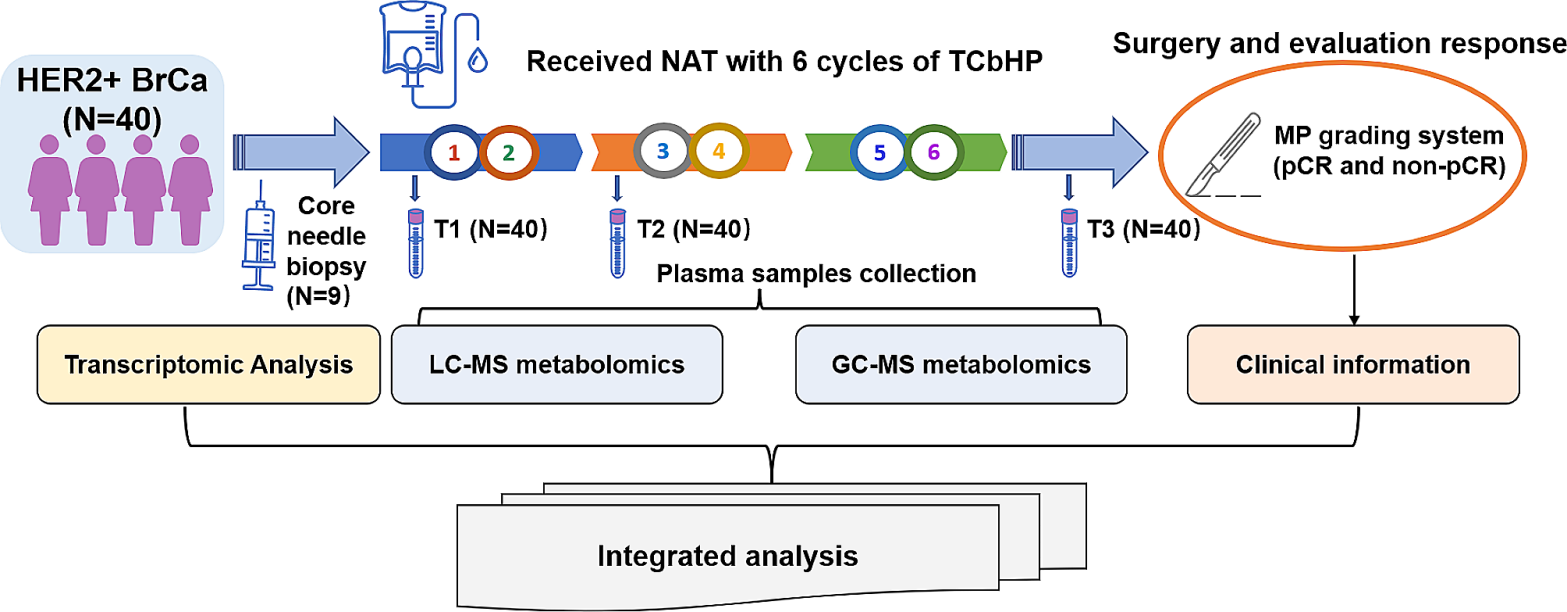
A schematic diagram of sample collection in the context of neoadjuvant therapy (NAT) followed by LC-MS, GC-MS metabolomics, transcriptomic RNA sequencing (RNA-seq), and integrated data analyses. T1, the time point at baseline of NAT. T2, the time point after 2 cycles of NAT. T3, the time point after 6 cycles (before surgery) of NAT. BrCa, breast cancer. pCR, pathologic complete response

Figure 1 shows the study workflow. A total of 40 HER2 + BrCa patients were eligible and recruited for this prospective study. Of these patients, 21 (52.5%) patients achieved pCR and 19 (47.5%) patients achieved non-pCR. The detailed demographic and clinicopathological characteristics of the participants are listed in Table 1. Patients in pCR and non-pCR groups exhibited no significant differences in baseline clinicopathological features including age at diagnosis, menopausal status, tumor laterality, T and N stage, ER and PR status, and the expression level of Ki67.

**Table 1:** Patient characteristics according to pathological response

Characteristics	All (*n* = 40) Number (%)	pCR (*n* = 21) Number (%)	Non-pCR (*n* = 19) Number (%)	*P*-value
**Age at diagnosis (median, y)**	50.5	51	50.0	0.935
≥ 50	21 (52.5)	11 (52.4)	10 (52.6)	0.987
< 50	19 (47.5)	10 (47.6)	9 (47.4)	
**Menopausal status**				0.516
Pre-	21 (52.5)	10 (47.6)	11 (57.9)	
Post-	19 (47.5)	11 (52.4)	8 (42.1)	
**Laterality**				0.726
Left	18 (45.0)	10 (47.6)	8 (42.1)	
Right	22 (55.0)	11 (52.4)	11 (57.9)	
T stage*				0.105
cT1	1 (2.5)	0 (0)	1 (5.3)	
cT2	21 (52.5)	9 (42.9)	12 (63.1)	
cT3	9 (22.5)	5 (23.8)	4 (21.1)	
cT4	9 (22.5)	7 (33.3)	2 (10.5)	
**N stage#**				0.666
cN1	14 (35.0)	8 (38.1)	6 (31.6)	
cN2	18 (45.0)	8 (38.1)	10 (52.6)	
cN3	8 (20.0)	5 (23.8)	3 (15.8)	
**ER**				0.115
Positive	29 (72.5)	13 (61.9)	16 (84.2)	
Negative	11 (27.5)	8 (38.1)	3 (18.8)	
**PR**				0.119
Positive	18 (45.0)	7 (33.3)	11 (57.9)	
Negative	22 (55.0)	14 (66.7)	8 (42.1)	
**Ki67**				1.000
≥ 20	35 (87.5)	18 (85.7)	17 (89.4)	
< 20	5 (12.5)	3 (14.3)	2 (10.5)	

*Notes* * cT1–2 vs. cT3–4; # cN1 vs. cN2-3

### GC-MS/LC-MS metabolomic analysis

#### Screening and identification of differential metabolites

**Fig. 2:**
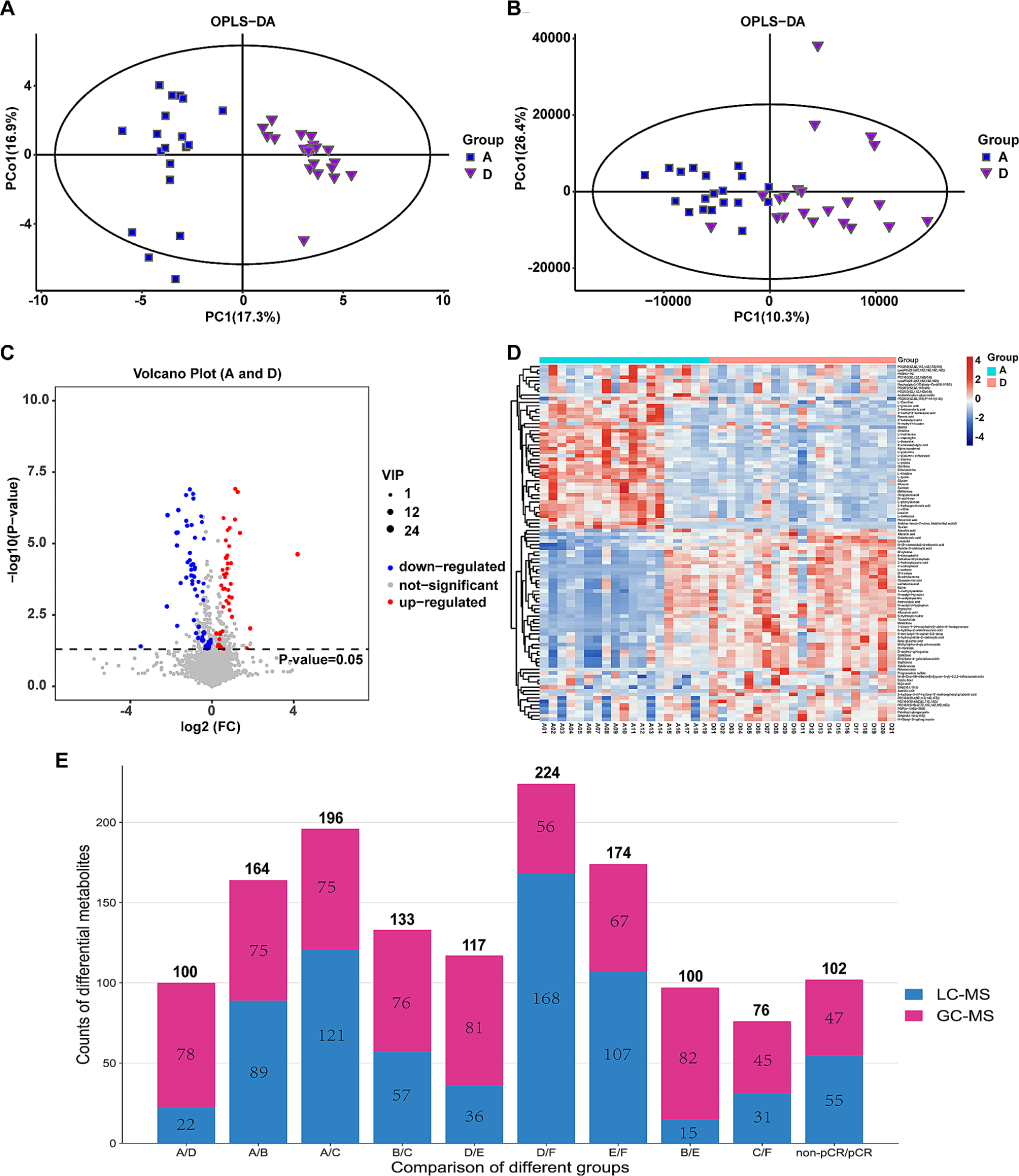
Analysis of multivariate data from LC-MS and GC-MS data. (**A**) The plot of the OPLS-DA score for the GC-MS data from non-pCR and pCR groups at the time point of baseline. (**B**) The plot of the OPLS-DA score for the LC-MS data from non-pCR and pCR groups at the time point of baseline. (**C**) Volcano diagram depicting the differentially expressed metabolites (DEMs) in the non-pCR and pCR groups at the time point of baseline. DEMs with VIP values higher than 1.0 and p-values lower than 0.05 were considered significant. Red and blue dots indicate up- and down-regulated metabolites, respectively. (**D**) Heatmap depicting the 100 pre-therapeutic DEMs between non-pCR patients and pCR groups according to metabolite class. Each column represents a subject and each row represents a metabolite. (**E**) The distribution of the counts of DEMs among the different groups. Group A, B, C, non-pCR patients, *N* = 19; group D, E, F, pCR patients, *N* = 21. Group A and D, at the time point of baseline; Group B and E, at the time point 2 cycles of neoadjuvant treatment; Group C and F, at the time point after 6 cycles (before surgery) of neoadjuvant treatment

GC-MS and LC-MS respectively detected 328 and 9536 peaks in positive and negative ionization modes. After the standing data, 305 and 3324 metabolites were identified in the 120 serum samples by GC-MS and LC-MS, respectively. OPLS-DA, which is commonly used to maximize the variances between groups in metabolomics analysis and identify metabolites with significant contribution to the variances [32–34], was used to gain further insights into the metabolomic profiles. Volcano plots were visualized using metabolites with VIP > 1.0 and *P* < 0.05. The results of OPLS-DA (Fig. 2A-B, Supplementary Figs. 1 and 2) and volcano plot analyses (Fig. 2C, Supplementary Fig. 3A and 3B) reveal significant differences in the metabolic features among the different groups. To completely and clearly illustrate the link between samples and the variations in metabolite expression between pCR group and non-pCR groups, we carried out a hierarchical clustering analysis based on the expression of all substantially varied metabolites. As shown in Fig. 2D, Supplementary Fig. 3C and 3D, compared with those in pCR groups, the metabolites in non-pCR groups were also significantly different, which may be related to the treatment response to NAT of the TCbHP regimen.

Figure 2E further shows the distribution of the counts of different metabolites among the different groups, and the detail information on the DEMs could be found in Supplementary Table 2. The data demonstrated that both in the non-pCR and pCR groups, the metabolic differences were most obvious after 6 cycles of NAT (A/C and D/F), and the total differential metabolites count was 196 and 224, respectively. The main objective of this investigation was to identify predictive markers of treatment efficacy, we focused on the analysis of DEMs between non-pCR and PCR groups at baseline with NAT treatment. The comparison at T1 time point revealed that, when comparing the non-pCR group with the pCR group (A/D), one hundred metabolites exhibited statistically significant changes, while the level of 46 metabolites were significantly upregulated, and 54 metabolites were significantly downregulated in non-pCR group. Among these 100 DEMs, 22 and 78 DEMs were identified via LC-MS and GC-MS, respectively.

#### Analysis of differential metabolites and metabolic pathways

**Fig. 3:**
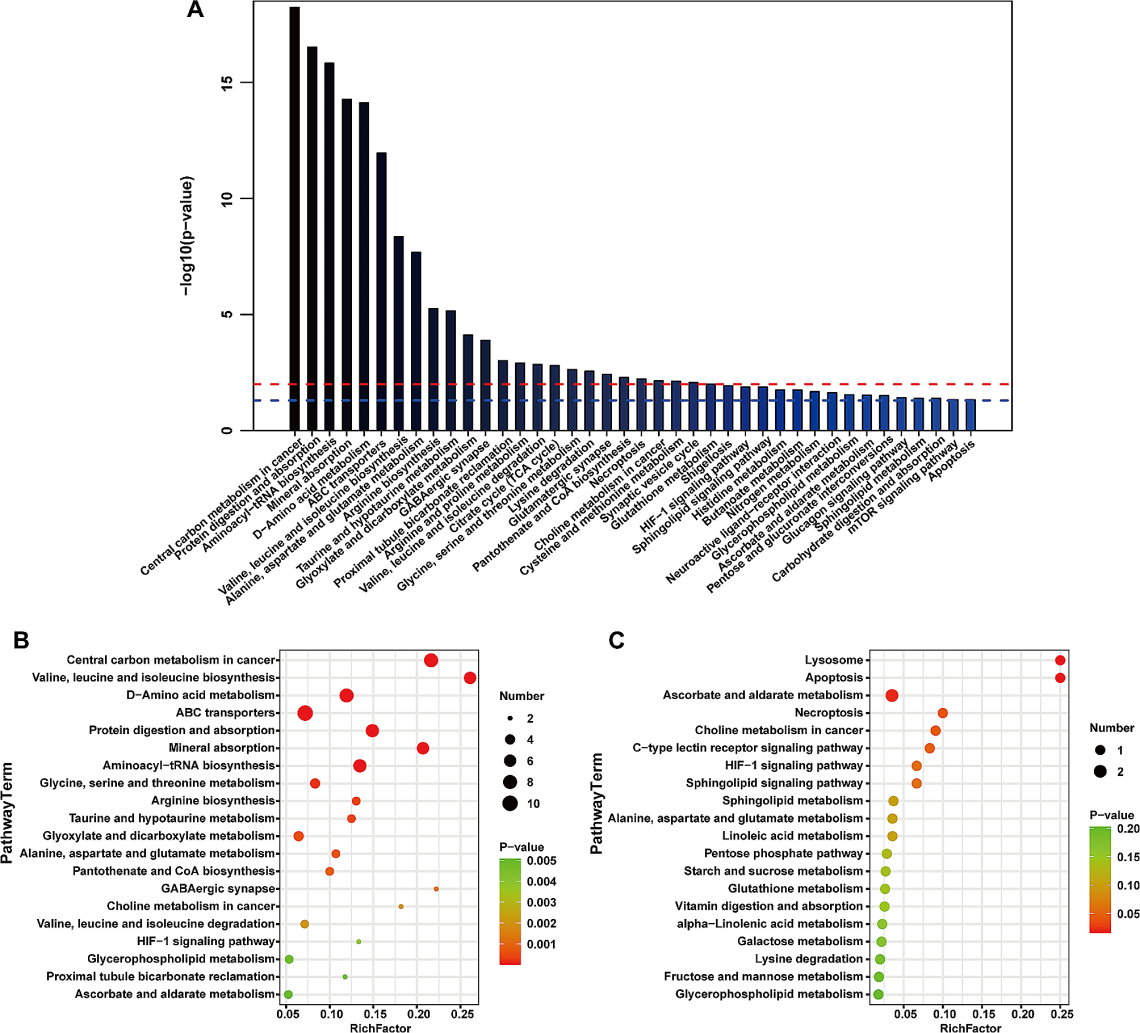
Metabolite pathway analysis using KEGG basing on pre-therapeutic DEMs. (**A**) Pre-therapeutic metabolic pathway enrichment of 100 differential metabolites between non-pCR patients and pCR patients at baseline of neoadjuvant therapy. The *P*-value of the red dashed line was 0.01 and that of the blue dashed line was 0.05. (**B**) Bubble plot depicting the KEGG pathways (top 20) in which 46 elevated metabolites were substantially enriched (*P* < 0.05); (**C**) Bubble plot depicting the KEGG pathways (top 20) in which 54 downregulated metabolites were substantially enriched (*P* < 0.05)

To further identify and understand the biological significance of these 100 DEMs between the A and D groups, the KEGG database was used to evaluate any possible biological significance. There were 40 metabolic pathways, and their *P* values were all significantly less than 0.05 (Fig. 3A). The most common enrichment factors were related to central carbon metabolism in cancer (glycine, L-alanine, L-asparagine, L-glutamic acid, L-glutamine, L-histidine, L-isoleucine, L-methionine, L-phenylalanine, L-proline, L-valine, leucine, oxoglutaric acid, pyruvic acid), protein digestion and absorption, aminoacyl-tRNA biosynthesis, mineral absorption, D-amino acid metabolism, and ABC transporters, which may greatly contribute to the therapeutic efficacy of TCbHP treatment. The top 20 significantly differentiated metabolic pathways of the upregulated and downregulated metabolites were shown in Fig. 3B and C, respectively. Obviously, upregulated and downregulated metabolites enriched pathways were different. Central carbon metabolism in cancer, valine, leucine and isoleucine biosynthesis, D-Amino acid metabolism were the top three enriched pathways of these 46 upregulated metabolites, while Lysosome, apoptosis, ascorbate and aldarate metabolism were the top three enriched pathways of these 54 downregulated metabolites. These results indicated that these altered metabolites and pathways might be associated with treatment response.

#### Screening of key differential metabolites

**Fig. 4:**
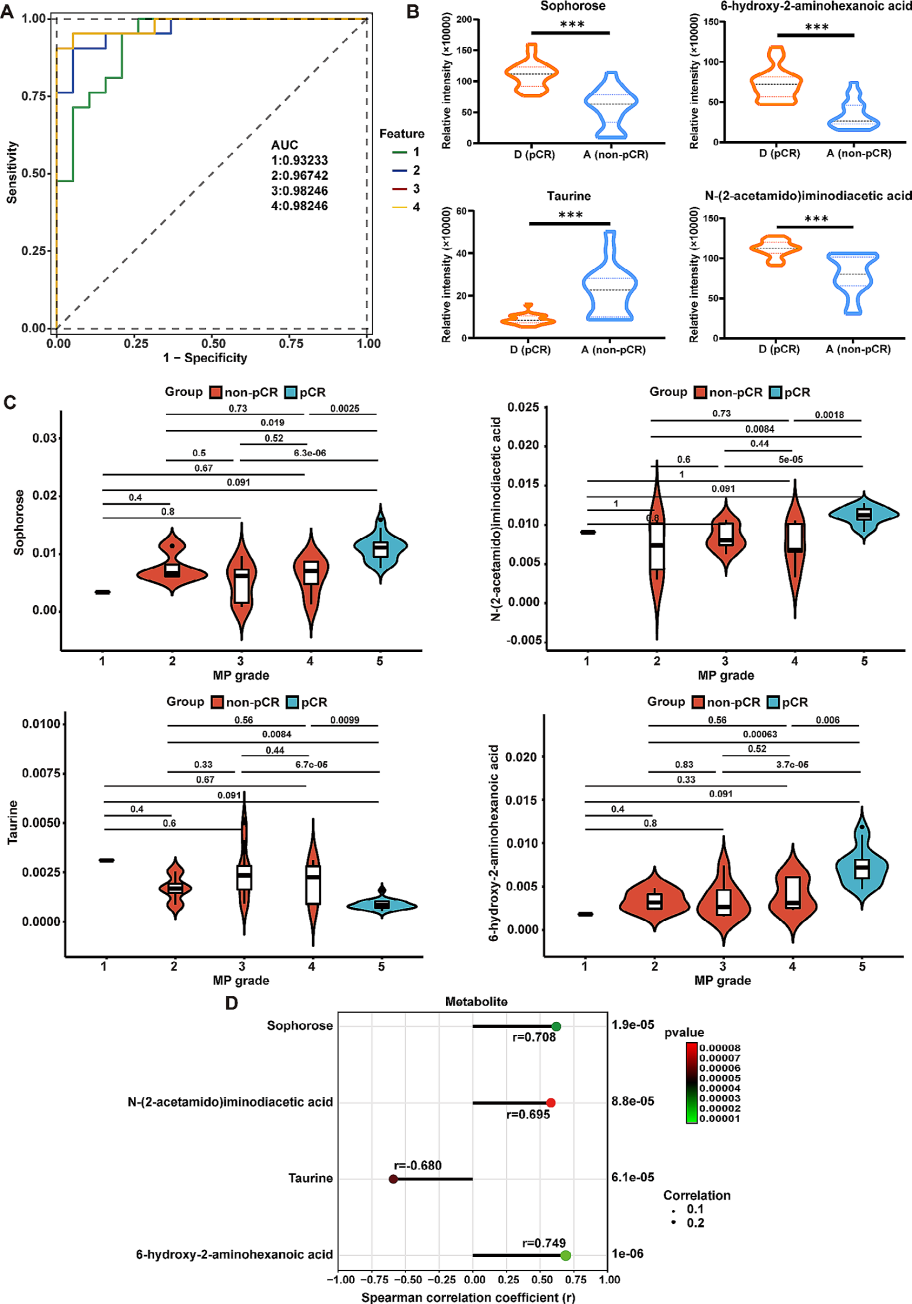
Comprehensive analyses of four pre-therapeutic metabolites performed via random forest analysis. (**A**) Receiver operating characteristic curve analysis of the efficacy of the four metabolites-based predictive model. 1: Sophorose; 2: Sophorose + ADA; 3. Sophorose + ADA + Taurine; 4: Sophorose + ADA + Taurine + 6-hydroxy-2-aminohexanoic acid; (**B**) Violin plots depicting the levels of four pre-therapeutic metabolites between non-PCR and PCR patients. (**C**) Violin plots depicting the levels of four pre-therapeutic metabolites among patients with different MP scores. (**D**) Spearman correlation of the four pre-therapeutic metabolites with MP grade

To identify the key metabolites associated with the treatment efficacy, the RF classifier was further performed based on these 100 pre-therapeutic DEMs that were compared between non-pCR and pCR groups (A/D). Four key metabolites [sophorose, N-(2-acetamido)iminodiacetic acid (ADA), taurine, and 6-hydroxy-2-aminohexanoic acid] were selected by RF analysis. The predictive accuracy of these four metabolites and the combined panel of these metabolites were evaluated for their ability to predict pCR. The AUC for the single metabolite of sophorose, ADA, taurine, and 6-hydroxy-2-aminohexanoic acid reached 0.932 (95% CI 0.858-1.000), 0.927 (95% CI 0.850-1.000), 0.910 (95% CI 0.822–0.998) and 0.935 (95% CI 0.859-1.000), respectively. The AUCs for sophorose + ADA, sophorose + ADA + taurine, and sophorose + ADA + taurine + 6-hydroxy-2-aminohexanoic acid were 0.967 (95% CI 0.922-1.000), 0.982 (95% CI 0.950-1.000) and 0.982 (95% CI 0.950-1.000), respectively (Fig. 4A). Among these metabolites, the concentrations of sophorose, ADA and 6-hydroxy-2-aminohexanoic acid were significantly higher in pCR group than in non-pCR group and were positively correlated with the MP grade. However, the taurine concentration was significantly higher in non-pCR group than in pCR group and was negatively correlated with the MP grade at baseline (Fig. 4B-D). These findings indicated that these four metabolites may be potential biomarkers for distinguishing non-sensitive individuals from sensitive individuals.

**Fig. 5:**
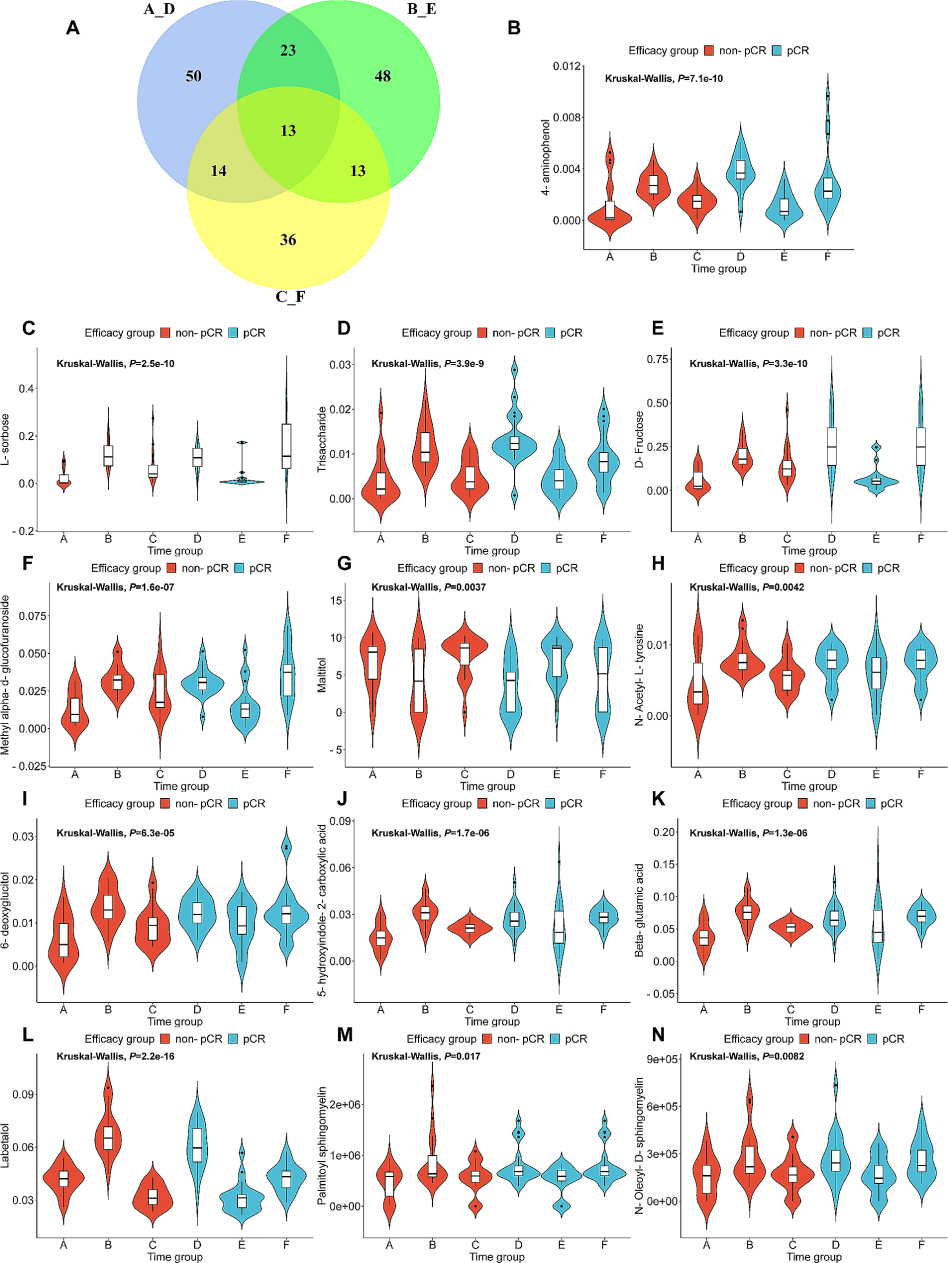
Thirteen metabolites exhibited permanent changes between non-pCR patients and pCR patients during the therapy term. (**A**) Venn plot was performed to identify metabolites that were persistently and differentially expressed between non-pCR patients and pCR patients (A vs. D, B vs. E and C vs. F). (**B**-**N**) Violin plots depicting the levels of 13 metabolites between non-pCR and pCR groups at different treatment time points. The Kruskal Wallis test was used to compare the metabolite expression difference among multiple groups

The metabolic profiles of the three-time points from which the serum samples were collected differed noticeably, indicating that the metabolome was altered during the course of NAT. To identify the metabolites related to treatment response over time, the Venn diagram was drawn for the DEMs in the A/D, B/E and C/F groups (Fig. 5A). Persistent differences of 13 metabolites, including 4-aminophenol, L-sorbose, trisaccharide, D-fructose, methyl alpha-d-glucofuranoside, maltitol, N-acetyl-l-tyrosine, 6-deoxyglucitol, 5-hydroxyindole-2-carboxylic acid, beta-glutamic acid, labetalol, palmitoyl sphingomyelin and N-oleoyl-D-sphingomyelin, were found during the treatment time. Among these metabolites, only maltitol was more abundant at the time point of T1, less abundant at T2, and more abundant at T3 in the non-pCR group than in the pCR group. However, the other 12 metabolites showed the opposite trend. The change curves over time are shown in Fig. 5B-N. These results indicated that these 13 metabolites could be potential biomarkers of residual disease.

**Fig. 6:**
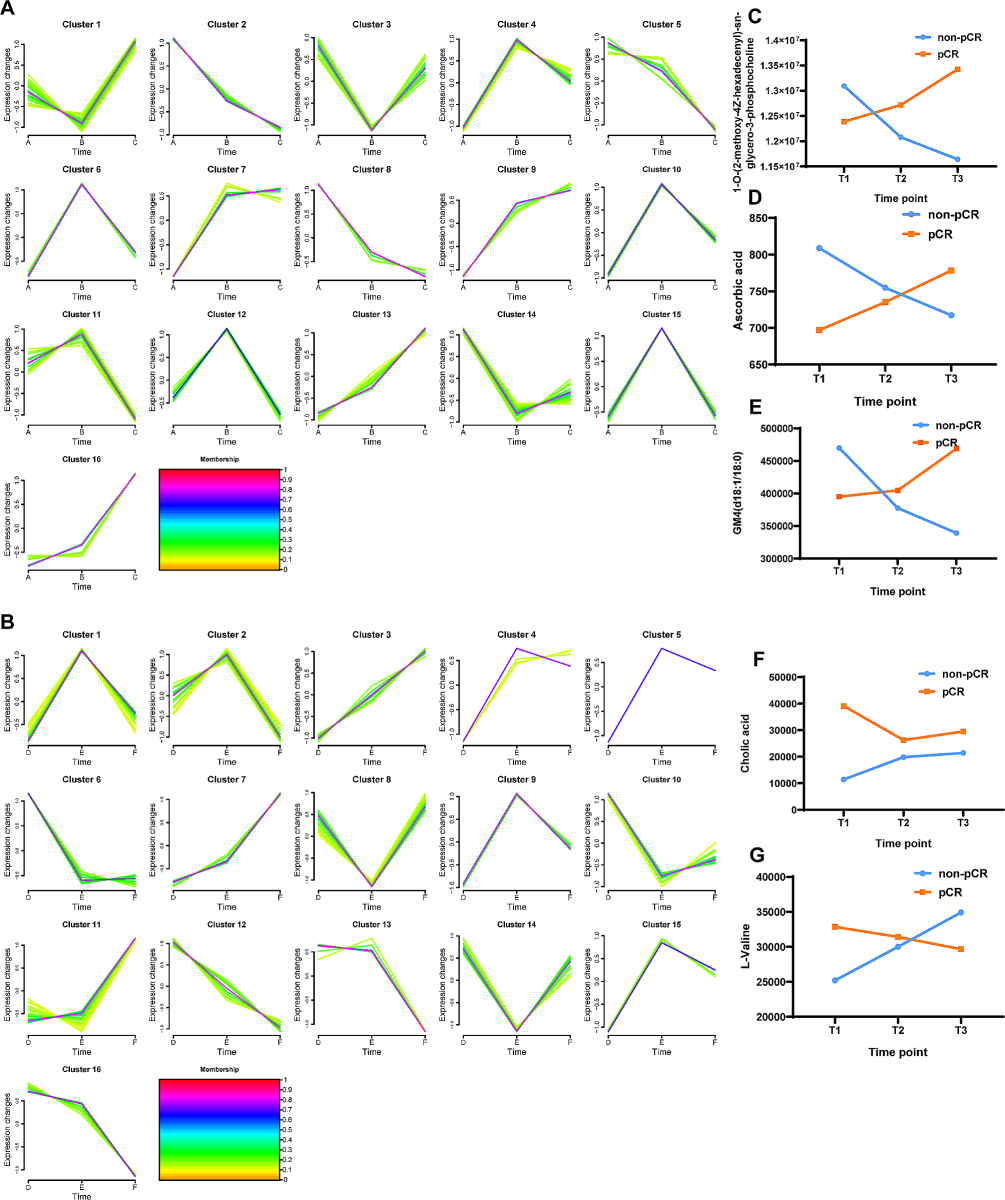
Dynamic alteration profile of metabolites in non-pCR and pCR patients basing on the total DEMs (102) in non-pCR and pCR patients. (**A**) Time trend cluster summary of serum metabolites in non-pCR group. (**B**) Time trend cluster summary of the serum metabolites in pCR group. Yellow or green lines represent metabolites with low membership values, whereas red and purple lines represent metabolites with high membership values. (**C**-**G**) Relative expression intensity of five metabolites that exhibited the same change trend over time in the same group. Each value of the metabolite is the mean of samples in every group at different time point. T1, the time point at baseline of neoadjuvant treatment. T2, the time point after 2 cycles of neoadjuvant treatment. T3, the time point after 6 cycles (before surgery) of neoadjuvant treatment

To understand the dynamic change profile during NAT, the total DEMs (102) in non-pCR and pCR patients were split into 16 groups (Fig. 6A-B) based on their trend similarity over time using the soft Mfuzz clustering algorithm. Clusters 2, 5, and 8 in non-pCR group and clusters 2, 6, 10, 12, 13, and 16 in pCR group trended to decrease over time. However, Clusters 7, 9, 13 and 16 in non-pCR group and clusters 3, 4, 7, 11, and 15 in pCR group trended to increase over time (Fig. 6A-B). Among these altered metabolites, 1-O-(2-methoxy-4Z-hexadecenyl)-sn-glycero-3-phosphocholine, GM4(d18:1/18:0), ascorbic acid, cholic acid and L-Valine might be the potential monitors for the treatment response (Fig. 6C-G). Comprehensive metabolomics showed that NAT therapy in HER2 + BrCa patients changed their metabolic profiles and identified sensitive metabolic features.

### Transcriptomic data analysis via RNA-seq

**Fig. 7:**
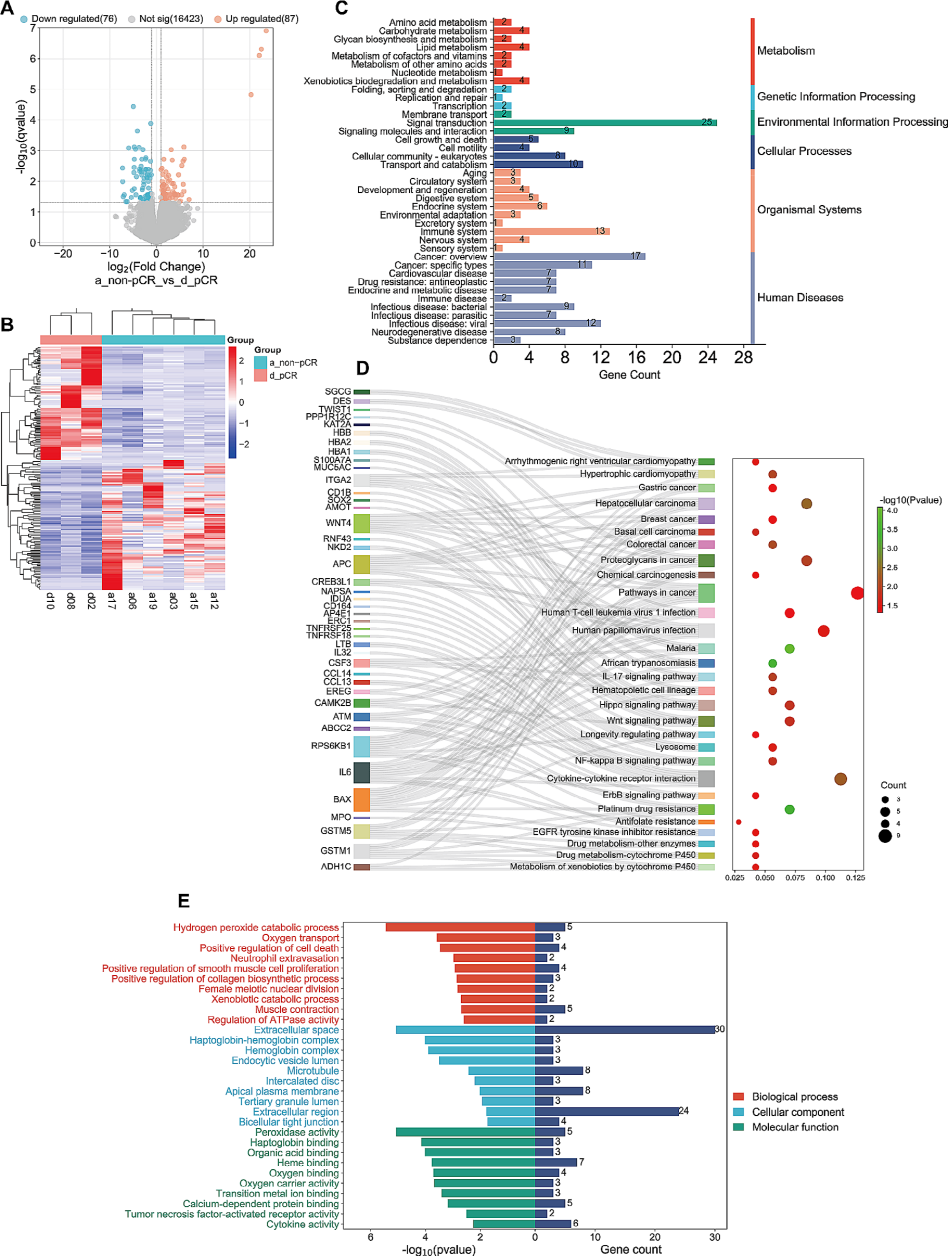
Identification of differentially expressed genes (DEGs) between non-pCR and pCR group and functional enrichment analysis for these DEGs. (**A**) Differential gene volcano plot (genes with q-value < 0.05 and |log2 fold change (FC)|>1 were considered as significant, red and blue dots indicate up- and down-regulated genes, respectively). (**B**) Cluster analysis of DEGs between non-pCR and pCR group. Each column represents a subject and each row represents a gene. (**C**) KEGG enrichment analysis of DEGs at level 1 and level 2. (**D**) Sankey dot plot for level 3 KEGG enrichment analysis of DEGs. (**E**) GO classification analysis of DEGs (top 30)

A total of 18,072 genes were identified in these nine biopsy tissue samples. Genes with q-value < 0.05 and |log2 fold change (FC)|>1 were considered as significant. 163 genes, including 87 upregulated and 76 downregulated genes, were identified as DEGs between non-pCR patients and pCR patients. As shown in Fig. 7A, the volcano plot demonstrated the overall distribution of DEGs. The two main hierarchical clusters based on the expression levels of DEGs are shown in Fig. 7B. Pathway analysis was also conducted to further functionally characterize the DEGs via using the KEGG database. KEGG enrichment analysis of the DEGs identified 6, 39 and 189 KEGG pathways at KEGG level 1, 2 and 3, respectively (Fig. 7C), signal transduction is the most enriched pathway of DEGs. The pathways with significant enrichment are shown in Fig. 7D, which illustrates the relationship between DEGs and pathways. In the GO enrichment analysis, 180 significantly abundant pathways were identified and the 30 most important pathways involved in cellular components, biological processes, and molecular functions are shown in Fig. 7E.

### Integration analyses of the transcriptomic and metabolomic data

**Fig. 8:**
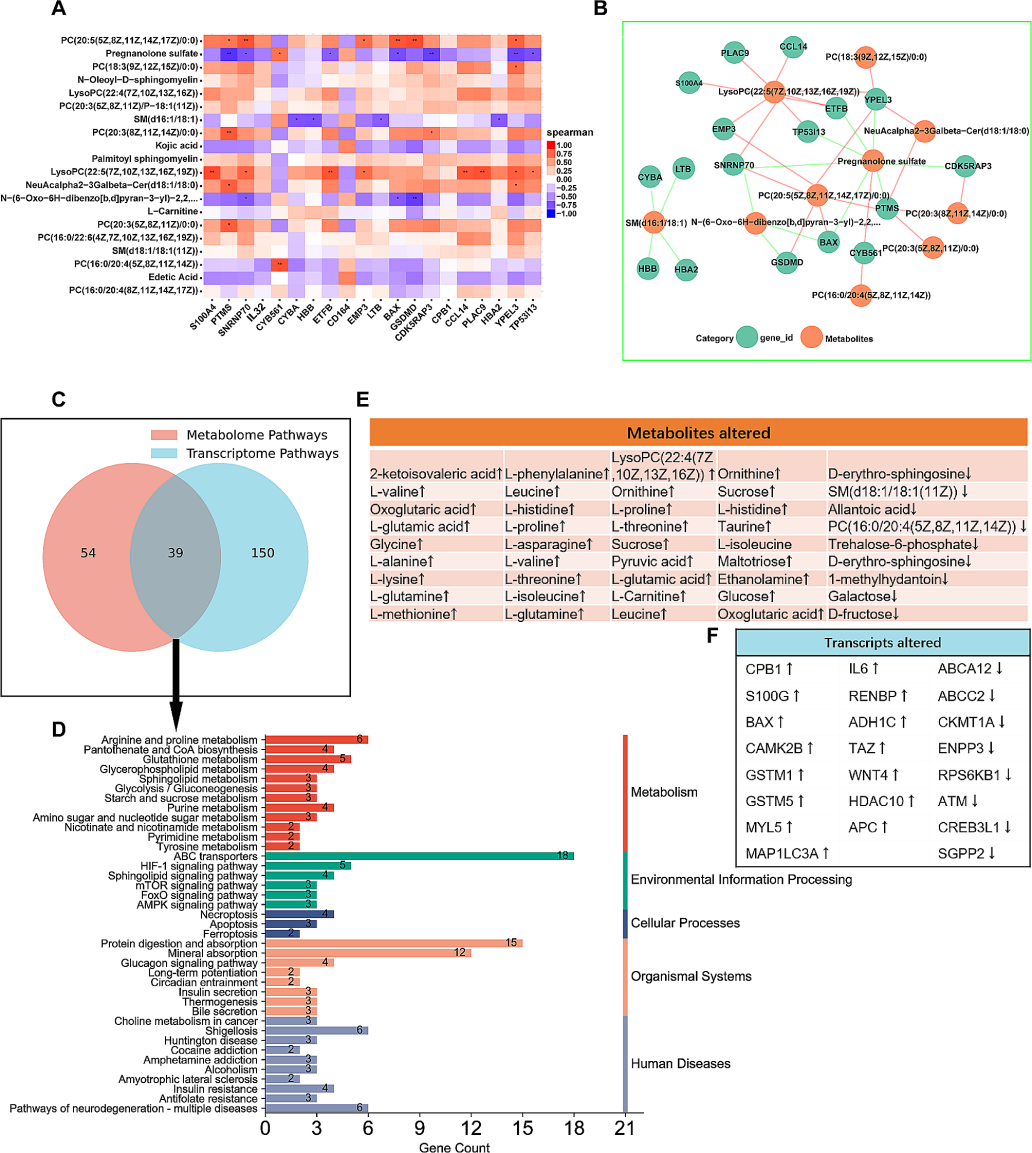
Integrated transcript and metabolite profile analysis. (**A**) Heatmap of the Spearman correlation coefficient matrix between the top 20 differentially expressed metabolites (DEMs) and the top 20 differentially expressed genes (DEGs). The red and blue colors represent a positive correlation and a negative correlation, respectively. The color label reflects the Spearman correlation coefficient value. (**B**) Cytoscape was used to construct the correlation network of the top 20 DEMs (red nodes) and the top 20 DEGs (green nodes). The results with *P* Value < 0.05 were shown. Positive and negative correlations are shown by the red and blue margins, respectively. (**C**)Venn diagram was used to identify the KEGG pathways in which both the DEMs and DEGs participated. (**D**) The histogram of KEGG pathways in which DEMs and DEGs are both enriched. (**E**) The DEMs involved in the integrated pathways. (**F**) The DEGs involved in the integrated pathways. DEMs were derived from 40 pre-therapeutic patients including 19 patients from non-pCR group and 21 patients from pCR group; DEGs were derived from nine patients including six patients from non-pCR group and three patients from pCR group. *P<0.05; ** *P*<0.01, ***P<0.001

Integrative analysis was performed to provide a more comprehensive understanding of the reason for the difference in treatment efficacy induced by the TCbHP regimen. First, Spearman correlations between the DEMs and DEGs were calculated pairwise to create a correlation network diagram based on the transcriptomic data from the nine patients and metabolomic data from the 40 pre-therapeutic patients. Figure 8A shows relationships between the top 20 DEMs and DEGs, and a significant (*P* < 0.05) transcript-metabolite interaction network was generated accordingly (Fig. 8B). Among them, several metabolites, such as LysoPC(22:5(7Z,10Z,13Z,16Z,19Z)) (VIP = 2.85, *P* value = 0.01469, Fold Change = 1.31) and PC(20:5(5Z,8Z,11Z,14Z,17Z)/0:0) (VIP = 1.20, *P* value = 0.04885, Fold Change = 1.48), were respectively markedly correlated with several of the top up-regulated DEGs, including S100A4, PLAC9, ETFB, CCL14, and GSDMD, SNRNP70, and BAX. However, pregnanolone sulfate (VIP = 1.67, P value = 0.02066, Fold Change = 0.62) was negatively correlated with up-regulated DEGs including PTMS, CDK5RAP3, and YPEL3. In addition, a joint analysis of the omics data at the pathway level revealed that 39 pathways were enriched on account of DEGs and DEMs (Fig. 8C). The pathways shared by the DEGs and DEMs mainly belonged to ABC transporters (glycine, L-alanine, L-glutamic acid, L-glutamine, L-histidine, L-isoleucine, L-lysine, L-phenylalanine, L-proline, L-threonine, L-valine, Leucine, maltotriose, ornithine, sucrose, taurine; ABCA12, ABCC2), protein digestion and absorption, mineral absorption, arginine and proline metabolism, shigellosis, pathways of neurodegeneration-multiple diseases, glutathione metabolism and the HIF-1 signaling pathway (Fig. 8D). Among the DEGs and DEMs related to the integrated pathways, 15 genes and 36 metabolites were respectively significantly upregulated, whereas 8 genes and 9 metabolites were significantly downregulated (Fig. 8E and F). Further correlation analysis of these DEGs and DEMs sharing common pathways demonstrated that 9 genes and 38 metabolites were significantly correlated (Supplementary Fig. 4A). In addition, in the KEGG functional annotation of DEGs, 13 genes were assigned to 8 metabolic pathways (Table 2), among which 7 genes were significantly correlated with 16 metabolites (Supplementary Fig. 4B). Overall, the integrated analysis successfully identified pathways and their related genes and metabolites that may affect the treatment efficacy of the TCbHP regimen.

**Table 2:** DEGs enriched in metabolic pathways

Classification_level2	Classification_level1	gene_number	percentage	Genes
Amino acid metabolism	Metabolism	2	2.82	ADH1C↑; CKMT1A↓
Carbohydrate metabolism	Metabolism	4	5.63	ADH1C↑; ENPP3↓; MTMR4↓; RENBP↑
Glycan biosynthesis and metabolism	Metabolism	2	2.82	IDUA↑; MAN2A1↓
Lipid metabolism	Metabolism	4	5.63	ADH1C↑; SGPP2↓; TAZ↑; TMEM86B↑
Metabolism of cofactors and vitamins	Metabolism	2	2.82	ADH1C↑; ENPP3↓
Metabolism of other amino acids	Metabolism	2	2.82	GSTM1↑; GSTM5↑
Nucleotide metabolism	Metabolism	1	1.41	ENPP3↓
Xenobiotics biodegradation and metabolism	Metabolism	4	5.63	ADH1C↑; GSTM1↑; GSTM5↑; MPO↓

### qRT-PCR detection results

Some DEGs were arbitrarily chosen for qRT-PCR detection to confirm the validity of the mRNA-seq data and to identify the probable genes implicated in trastuzumab resistance. These findings indicated that several DEGs, such as ABCA12, CKMT1A, CDK5RAP3, CYBA, DMAP1, HDAC10, LRFN4, MYL5, PPP1R12C, RENBP, and TP53I13 might be both associated with primary and acquired resistance of trastuzumab. Other DEGs, such as CPB1, GTF2I, HAUS7, IDUA, IL6, PLAC9, and TAZ might be indicators only for primary resistance but not for acquired resistance of trastuzumab (Supplementary Fig. 5).

## Discussion

In the present study, our data showed that, compared with non-pCR patients, pCR patients with HER2 + BrCa had unique metabolic features at baseline and during different treatment periods. These metabolic changes were most likely the result of many metabolic pathways being dysregulated. Importantly, we discovered four serum metabolites for predicting treatment response in patients receiving the TCbHP regimen, offering an opportunity to identify insensitive individuals before NAT. Further qRT-PCR detection results in cell lines indicated that several DEGs might be associated with trastuzumab resistance. Integrated metabolomics and transcriptomics data suggested that metabolites and transcripts were significantly correlated and involved in several common pathways, which indicated that these significant alterations might be associated with treatment response.

One of our main objectives was to explore metabolite-based biomarkers for the prediction of NAT response in HER2 + BrCa patients. Here, we selected prediction models using random forest. A set of 4 metabolites, sophorose, ADA, taurine and 6-hydroxy-2-aminohexanoic acid, was evaluated as new predictive indicators. The selected biomarkers have more accurately predicted ability for NAT efficacy, and the AUC of the constructed prediction model was greater than 0.932. However, studies with large-scale and longitudinal cohorts of NATs are needed to validate the potential biomarkers and substantiate these findings. The identification of these potential NAT response predicting metabolites could help to gain insight into the understanding of underlying mechanisms of drug resistance.

In the present study, sophorose was up-regulated in pCR group compared with non-pCR group. Sophorose is a disaccharide, a glucose dimer. It is distinct from other glucose dimers, such as maltose, because its β-1,2 bond is unique. It was isolated from sophora japonica stems in 1938 [35]. It is a component of sophorolipids and is a product of the caramelization of glucose [36]. The role of sophora in cancer is less well understood, but sophorolipids are gaining interest as potential cancer therapeutics due to their inhibitory effects on a range of cancer cells including those of the breast, cervical, colon, liver, brain, and pancreas [37]. Our study indicated that HER2 + BrCa patients with higher concentrations of sophorose may have better treatment responses to NAT with TCbHP, which is consistent with the anti-tumor effect of sophorolipids. ADA and 6-hydroxy-2-aminohexanoic acid were up-regulated in pCR group compared with non-pCR group. However, the specific roles of ADA and 6-hydroxy-2-aminohexanoic acid in cancer are currently limited.

Taurine (2-aminoethane-sulfonic acid) is a non-essential amino acid that is found in millimolar concentrations in most mammalian tissues [38]. Humans manufacture taurine endogenously but obtain it from food [39, 40], especially seafood. Taurine regulates cell volume, osmoregulation, membrane stability, bile salt conjugation, antioxidation, inflammation, and autophagy [38, 41, 42]. Agouza’s study revealed that BrCa patients had considerably lower serum taurine levels than high-risk BrCa patients and people with benign breast lesions [43]. However, other investigations have shown that serum taurine levels are higher in cancer patients including those with BrCa [44], endometrial cancer [45], and bladder cancer [46], than in controls. These inconsistent results indicate that further researches are needed to determine whether the serum taurine concentration is associated with tumor malignancy and whether taurine may serve as a metabolomic marker for the development of malignant tumors. In addition, taurine has an antitumor effect by increasing antioxidant capacity, boosting immunity, and triggering the death of tumor cells in BrCa [47, 48] and other types of cancer cells including glioblastoma [49], lung [50], colon [51], nasopharynx [52] and ovarian cancer [53]. Patients with lung cancer who have elevated serum taurine levels typically respond to PD-1 blockade antibody therapy [54]. After treatment with NAT, bladder cancer patients with upregulated serum taurine levels could easily achieve pCR [55]. Moreover, taurine combined with chemotherapeutic drugs, including cisplatin and doxorubicin, can increase the effectiveness of chemotherapeutic medications and lessen their side effects [40]. These findings suggested that taurine has the potential to improve the efficacy of immunotherapy or chemotherapy. However, in our study, the serum taurine concentration was higher in non-pCR group than in pCR group and was negatively correlated with MP grade. The analyses of the integration of previous studies and our unexpected results have given rise to several interesting questions that need to be addressed by further studies. First, whether taurine has an antitumor effect in HER2 + BrCa. Second, whether taurine was a true indicator for NAT response of TCbHP in HER2 + BrCa. Third, whether taurine influences the process of the biological effect or drug efficacy of the chemotherapeutic drugs (taxane and carboplatin) or targeted drugs (trastuzumab and pertuzumab) in the body. Fourth, whether dietary supplement taurine is reasonable for HER2 + BrCa patients receiving NAT.

No matter the pathway analyses of pre-therapeutic metabolic traits or integrated with transcriptional traits, the results confirmed that dysregulation of cancer-related metabolic pathways was also related to the treatment effect of TCbHP. The central carbon metabolismin in cancer was the top up-regulated pathway according to the metabonomic analysis. ABC transporters were one of the top 4 up-regulated pathways according to the metabonomic analysis and were the top dysregulated pathways in integrated analysis. The central carbon metabolism in cancer pathway involves aerobic glycolysis, increased glutaminolysis, a dysregulated TCA cycle, and the pentose phosphate pathway, which is the host’s primary source of energy [56]. In addition to being necessary for the proliferation of cancer cells, the central carbon metabolism in cancer plays a fundamental role in metabolic reprogramming and is vital for the function of endothelial cells, stromal cells, CTLs, regulatory T cells, and myeloid cells [18]. Changes in central carbon metabolism in the cancer pathway of cancer stem cells have been reported [57]. ABC transporters are one of the most well-known mechanisms of multidrug resistance, and they are involved in a variety of physiological processes such as cholesterol homeostasis, the transport of numerous chemicals into and out of cells and organelles, oxidative stress, immunological recognition, and drug efflux [58]. Notably, the ABC transporter pathway is required for lipid homeostasis, lipid trafficking, and signaling, which are essential functions for cell function [59]. Our findings showed that a range of substrates, including carbohydrates and amino acids, were also transported by this route. Changes in the biological pathways of these metabolites and genes may aid in understanding the possible mechanism underlying the therapeutic response of HER2 + BrCa patients to NAT.

In the present work, we collected samples at three different points during NAT to examine dynamic changes in metabolites and to investigate the metabolic processes involved in the NAT treatment response in HER2 + BrCa patients. To the best of our knowledge, no metabolomics studies have been conducted to investigate the alterations in metabolic patterns during NAT in HER2 + BrCa patients. This study offered an in-depth look at the metabolite alterations that occur during NAT. According to the dynamic metabolomics analyses, 13 metabolites exhibit persistent differences during the different periods of NAT. However, they showed an opposite trend at the second cycle of treatment compared with the time point at baseline and at the end of treatment. Another five metabolites exhibited the same changing tendency in the same group at different time points. The metabolome exhibits dynamic changes over time, which corresponds to the trajectory of developing illness [60]. Thus, we speculate that these metabolites might be related to the efficacy of treatment. However, further studies need to be conducted to evaluate whether these metabolites are ideal monitoring markers for NAT in HER2 + BrCa patients.

At present, in BrCa, metabolomics-based techniques have been employed in a wide range of applications, including screening, predicting therapy response, forecasting recurrence diagnosis, and evaluating prognosis [25]. A previous study revealed that the metabolomic profile of HER2 + participants who received lapatinib had substantial prognostic accuracy in terms of time to progression and overall survival [61]. Serum metabolites might be used as diagnostic biomarkers for HER2 + BrCa and could serve as predictors of trastuzumab therapy efficacy [62]. Other studies have shown that metabolomics can predict the response to NAT with epirubicin plus cyclophosphamide, followed by three weekly doses of docetaxel +/- trastuzumab [63]; or trastuzumab-paclitaxel [64]. In our study, we discovered substantial metabolic alterations in the serum as a response to therapy by metabolic profiling of blood samples taken before, during, and after NAT in HER2 + BrCa patients. This provides insight into how therapy affects the body and suggests that metabolomics might be a useful technique for identifying biomarkers to predict or monitor the treatment response in patients with TCbHP. In addition, the qRT-PCR results obtained from the trastuzumab-sensitive and trastuzumab-resistant cell lines were consistent with the expression patterns of the DEGs identified by RNA-seq, suggesting that some of the genes whose expression was altered might be involved in primary or acquired trastuzumab resistance. Moreover, the integration of metabolomic and transcriptomic data can help to clarify the processes that underlie the association between altered metabolite levels and treatment response. However, we should note that transcriptomic analysis has several inherent limitations. For example, relevant tissues are often unavailable, and this difficulty is not easily overcome. Moreover, RNA degradation is a serious limitation of transcriptomic studies. These shortcomings are very obvious in our study due to but not limited by the small size of the puncture tissue specimens and longer time span or longer sample preservation time before RNA-seq analysis. However, untargeted metabolomic investigations of serum metabolites are indicators of tumor/host metabolism, could reveal downstream risk factors, and have the advantages of rapid, non-invasive, relatively inexpensive, and facilitating dynamic monitoring; moreover, these methods are very suitable for identifying valuable biomarkers clinically. Our findings imply the potential clinical application of serum-metabolomics for predicting or monitoring treatment efficacy before or during NAT. Thus, we propose that metabolomics could be a promising approach for predicting or monitoring the sensitivity of NAT to the TCbHP regimen using significantly abnormally changed metabolites from serum samples. Moreover, the utility of transcriptomics could be used as a supplement to metabolomics in the discovery of biomarkers related to treatment efficacy, understanding of drug resistance mechanisms, and helping to explore potential intervention targets or strategies.

However, it is important to note that our study has several limitations. First, the sample size was quite small, which might lead to insufficient discovery and overlooking of other relevant indicators. Second, these identified metabolites were not further validated in a larger number of samples or external cohorts. Third, although we found that some genes may be associated with trastuzumab resistance, the NAT regimen used in this study included four different drugs, identifying a definite relationship between potential biomarkers or pathways with the resistance of a specific drug is difficult. Moreover, it is uncertain whether these identified biomarkers are also applicable to other neoadjuvant anti-HER2 therapy regimens, such as taxane and trastuzumab in combination with pertuzumab or pyrotinib, which are also recommended by guidelines and used in clinical practice. Fourth, the current study did not uncover the underlying molecular mechanism for why changes in the levels of these metabolites or genes are connected with medication sensitivity, which will be determined in future research.

## Conclusions

In summary, comprehensive utilization of non-targeted GC-MS and LC-MS metabolomics revealed metabolomic patterns linked with the therapeutic response to NAT with TCbHP regimen in HER2 + BrCa patients. Four potential new serum metabolic predictive indicators could be used to effectively discriminate TCbHP resistance from sensitivity, allowing for the early prediction of the treatment response to NAT with TCbHP in the HER2 + BrCa population. Significantly altered metabolic pathways could offer mechanistic insight into drug resistance and aid in the development of novel therapeutic targets for insensitive patients. Moreover, several abnormally altered metabolites may have potential value in monitoring the efficacy of treatment. Moreover, several abnormally expressed genes might be associated with trastuzumab resistance. Integrating metabolomics and transcriptomics could assist in obtaining new insights into biochemical pathophysiology and generating hypotheses for future research. Future research is needed to corroborate these findings in a large patient population and to investigate the resistance-mediating mechanism and their potential clinical applications in HER2 + BrCa patients.

### Electronic supplementary material

Below is the link to the electronic supplementary material.

Supplementary Material 1

Supplementary Material 2

Supplementary Material 3

## Data Availability

The datasets generated during the current study are available from the corresponding author on reasonable request.

## Abbreviations

BrCa: breast cancer
HER2+: human epidermal growth factor receptor 2 positive
T: trastuzumab
P: pertuzumab
T: taxane
Cb: carboplatin
NAT: Neoadjuvant therapy
pCR: pathologic complete response
LC-MS: liquid chromatography-mass spectrometer
GC-MS: gas chromatography-mass spectrometer
RSD: relative standard deviation
DEGs: differentially expressed genes
DEMs: differentially expressed metabolites
qRT-PCR: quantitative real-time polymerase chain reaction
ER: estrogen receptor
PR: progesterone receptor
ASCO: American Society of Clinical Oncology
CAP: College of American Pathologists
MRI: magnetic resonance imaging
CT: computed tomography
PET: positron emission tomography
AUC: area under curve
MP: Miller-Payne
QC: quality control
RIN: integrity number
FPKM: fragments per kilobase million
NB: negative binomial distribution
PCA: principal component analysis
OPLS-DA: orthogonal partial least squares discriminant analysis
RPT: response permutation testing
VIP: Variable importance of projection
KEGG: Kyoto Encyclopedia of Genes and Genomes
GO: Gene Ontology
ROC: receiver operating characteristic
ADA: N-(2-acetamido)iminodiacetic acid
