# Supplementary material for: Metabolomics assisted by transcriptomics analysis to reveal metabolic characteristics and potential biomarkers associated with treatment response of neoadjuvant therapy with TCbHP regimen in HER2 + breast cancer
Source: Breast Cancer Res. 2024 Apr 12;26:64. doi: 10.1186/s13058-024-01813-w (PMC11010353; doi:10.1186/s13058-024-01813-w)

**Supplementary Figure 1.** **The OPLS-DA score plot of the GC-MS data for different groups.** Group A, B, C, non-pCR patients, N=19; group D, E, F, pCR patients, N=21. Group A and D, at the time point of baseline; Group B and E, at the time point 2 cycles of neoadjuvant treatment; Group C and F, at the time point after 6 cycles (before surgery) of neoadjuvant treatment.


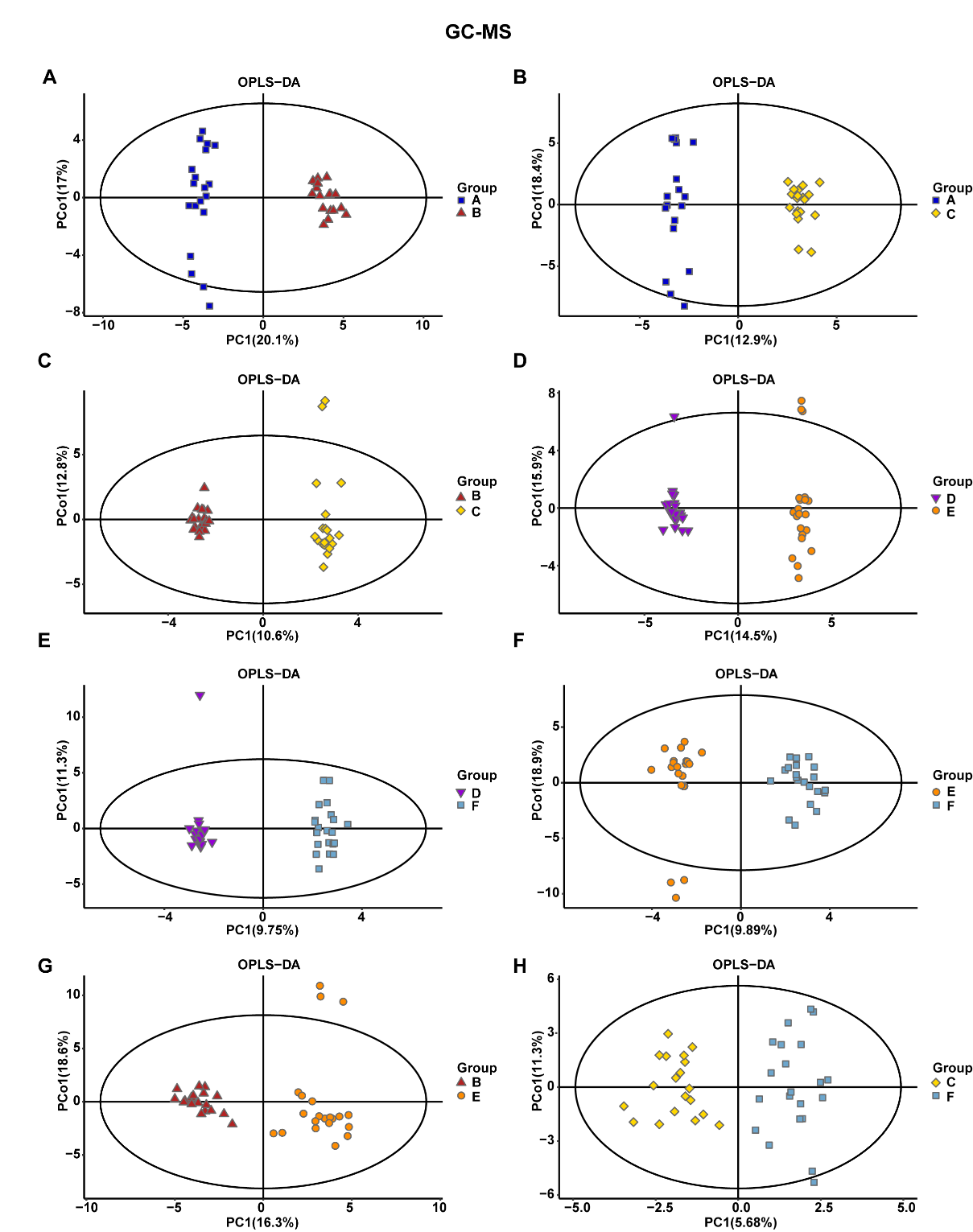


**Supplementary Figure 2.** **The OPLS-DA score plot of the LC-MS data for different groups.** Group A, B, C: non-pCR patients, N=19; group D, E, F: pCR patients, N=21. Group A and D, at the time point of baseline; Group B and E, at the time point 2 cycles of neoadjuvant treatment; Group C and F, at the time point after 6 cycles (before surgery) of neoadjuvant treatment.


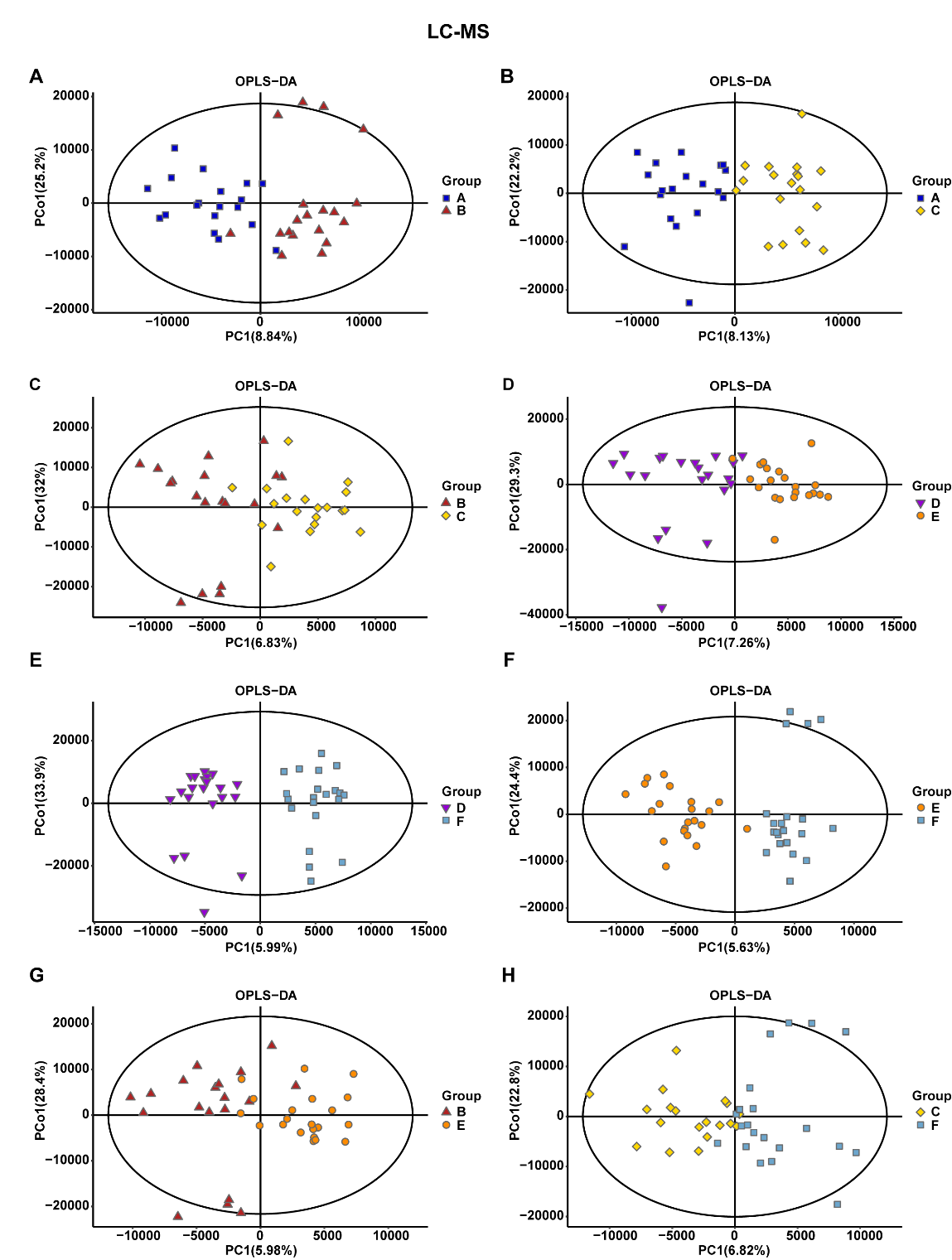


**Supplementary Figure 3. Volcano diagram and Heatmap for the differences in metabolites between non-pCR patients and pCR patients.** (A) Volcano diagram depicting the differentially expressed metabolites in the non-pCR and pCR groups at the time point of 2 cycles after treatment (group B: non-pCR patients, N=19; group E: pCR patients, N=21). (B) Volcano diagram depicting the differentially expressed metabolites in the non-pCR and pCR groups at the time point of 6 cycles after treatment (group C: non-pCR patients, N=19; group F: pCR patients, N=21). DEMs with VIP values higher than 1.0 and *P* values lower than 0.05 were considered as significant. Red and blue dots indicate up- and down-regulated metabolites, respectively. (C) Heatmap depicting the differences in metabolites between non-pCR patients and pCR patients according to metabolite class at the time point of 2 cycles after treatment (group B: non-pCR patients, N=19; group E: pCR patients, N=21). (D) Heatmap depicting the differences in metabolites between non-pCR patients and pCR patients according to metabolite class at the time point of 6 cycles after treatment (group C: non-pCR patients, N=19; group F: pCR patients, N=21). Each column represents a subject and each row represents a metabolite.


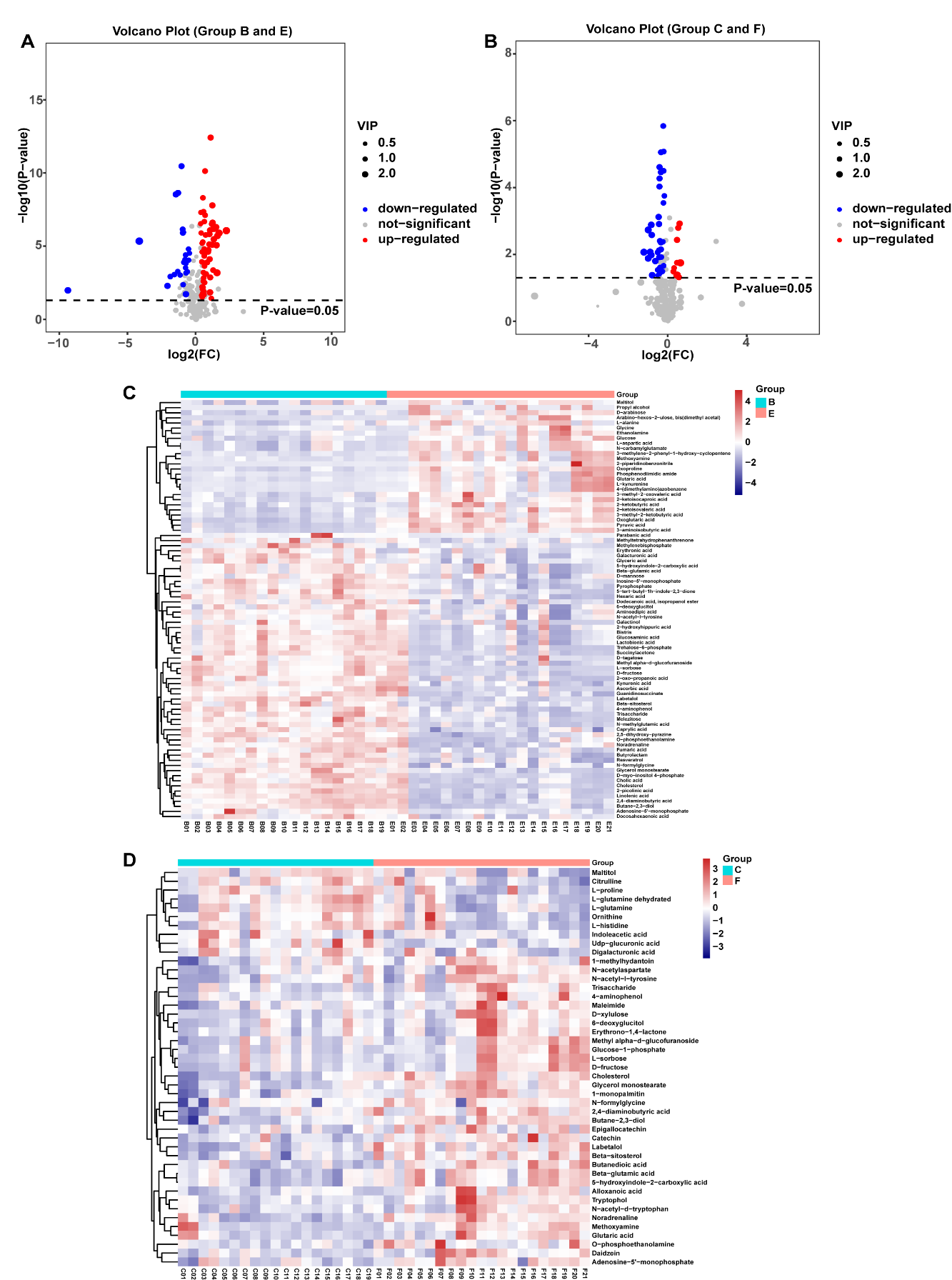


**Supplementary Figure 4.** Spearman correlation analyses of several differentially expressed genes (DEGs) and several differentially expressed metabolites (DEMs) obtained from the integration analysis of the transcriptomic and metabolomic data. (A) Heatmap of the correlation matrix between DEGs and DEMs with shared pathways. (B) Heatmap of the correlation coefficient matrix between metabolic pathway-enriched DEGs and DEMs. DEMs were derived from 40 pre-therapeutic patients including 19 patients from non-pCR group and 21 patients from pCR group; DEGs were derived from nine patients including six patients from non-pCR group and three patients from pCR group. **P*＜0.05; ** *P*＜0.01, ****P*＜0.001.


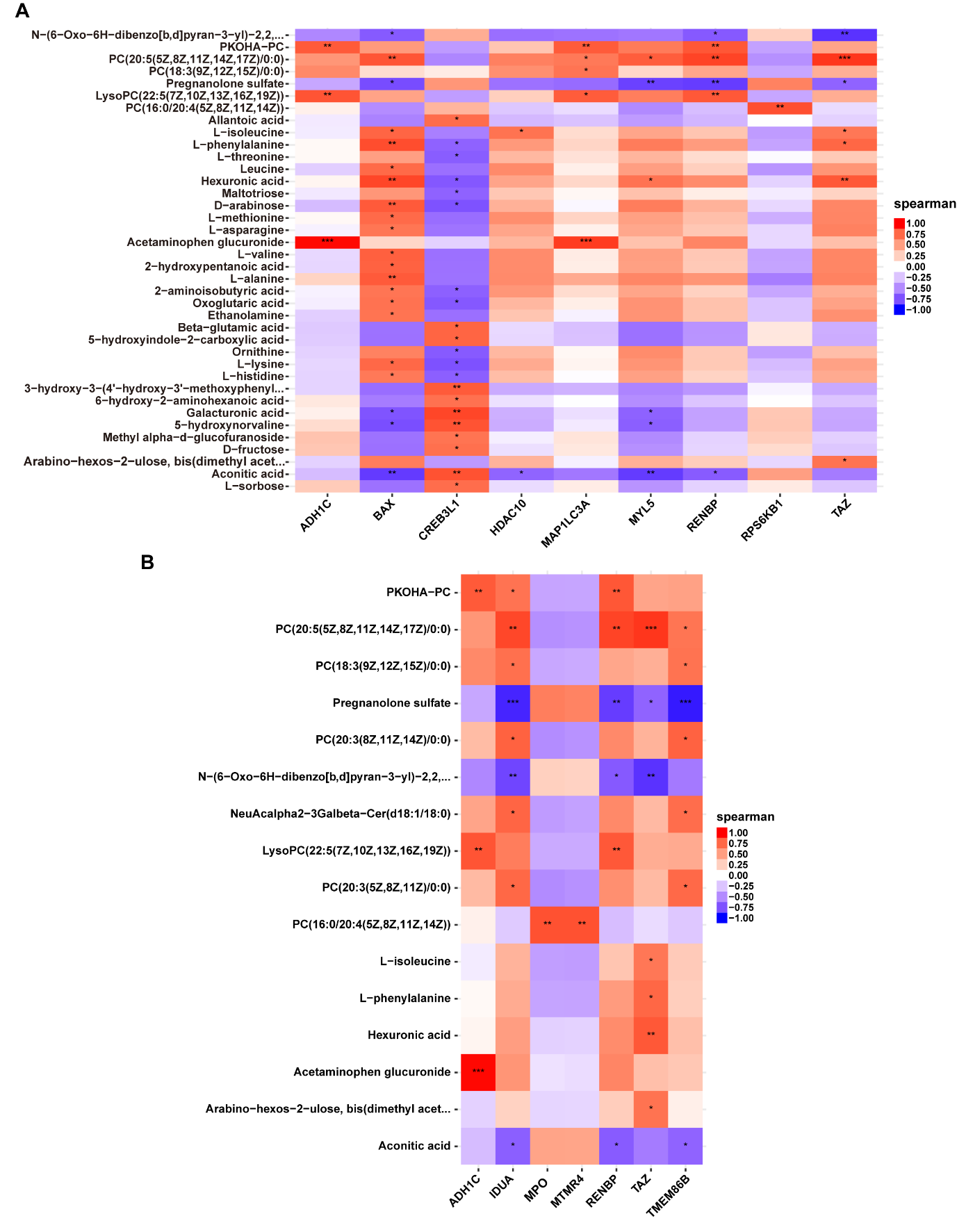


**Supplementary Figure 5.** qRT-PCR results for several differentially expressed genes (DEGs) in trastuzumab-resistant and trastuzumab-sensitive cell lines screened by RNA-seq in tissues from six patients in the non-pCR group (a03, a06, a12, a15, a17, a19) and three patients in pCR group (d02, d08, d10). The relative level of mRNA expression was normalized to that of β-actin in each sample, and values are means ± SD (n = 3). **P*＜0.05；** *P*＜0.01, ****P*＜0.001. Student t test was used to generate *P* value.


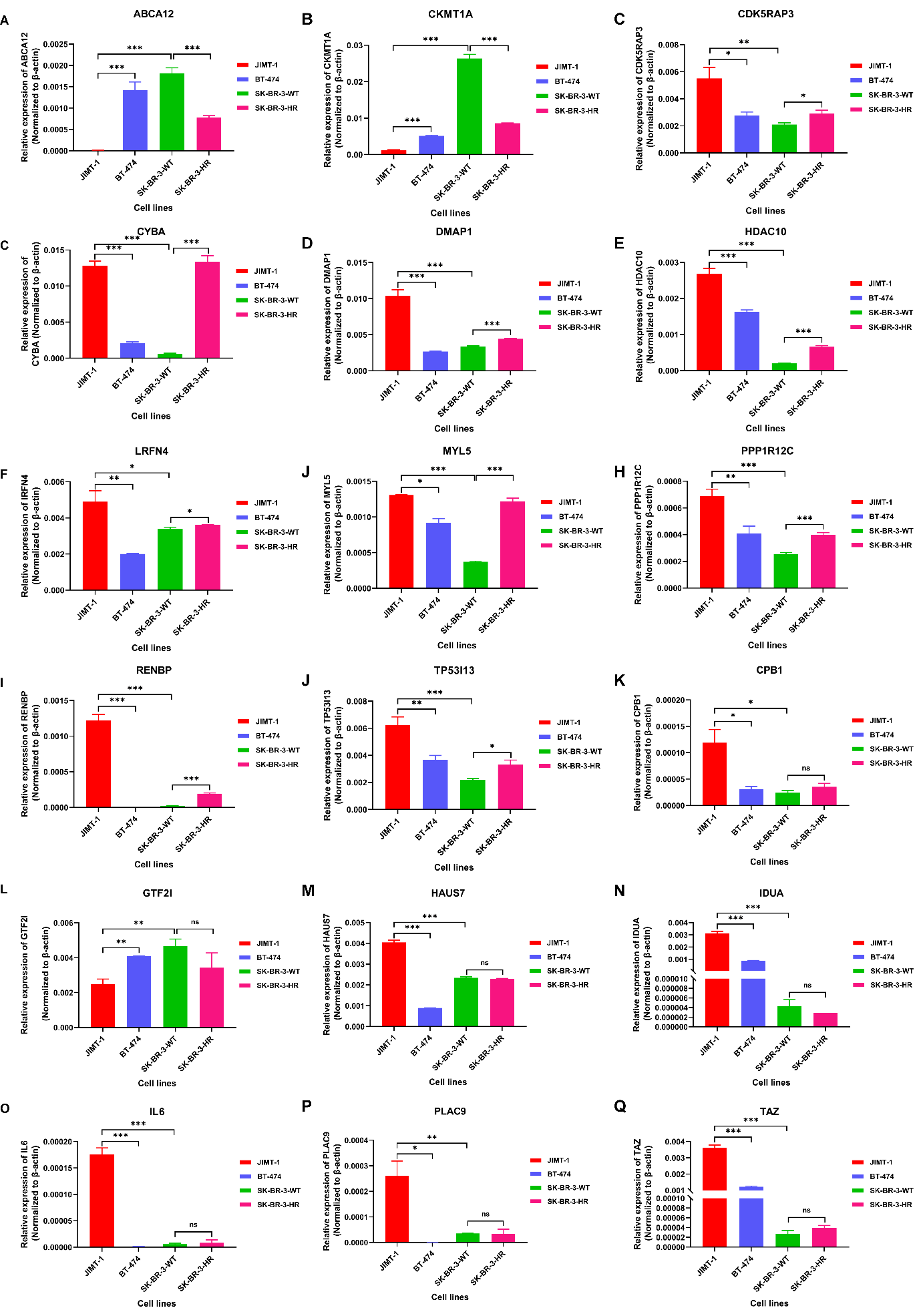

Supplement: Supplementary file 1 — Supplementary Material 1 [file 13058_2024_1813_MOESM1_ESM.docx]
