# Supplementary material for: Metabolomics assisted by transcriptomics analysis to reveal metabolic characteristics and potential biomarkers associated with treatment response of neoadjuvant therapy with TCbHP regimen in HER2 + breast cancer
Source: Breast Cancer Res. 2024 Apr 12;26:64. doi: 10.1186/s13058-024-01813-w (PMC11010353; doi:10.1186/s13058-024-01813-w)
Supplement: Supplementary file 2 — Supplementary Material 2 [file 13058_2024_1813_MOESM2_ESM.docx]

**Supplementary Table 1. The primers and their sequences utilized in the present study.**

| **Genes** | **Forward** | **Reverse** |
| --- | --- | --- |
| β-actin | CATGTACGTTGCTATCCAGGC | CTCCTTAATGTCACGCACGAT |
| ABCA12 | TGGACGAGGCTGAAGTG | TTGGTAAGCGTGAGGTGAT |
| CKMT1A | CATGGCTGGTCCCTTCTC | CCTCCGTCGTTCACTGG |
| CDK5RAP3 | GAGTCTGGTGCTGACGATCC | TGTGAAGAGTATCGGCCAAAAAT |
| CYBA | CCCAGTGGTACTTTGGTGCC | GCGGTCATGTACTTCTGTCCC |
| DMAP1 | CCACGATGATGCTTGGACTAAG | GATGTGGTAGTACCGCTCCTT |
| HDAC10 | CCAACAGGAAGCGTCAGC | CCTGCCCATCCAGCATC |
| LRFN4 | GACCATAACCTTATTGACGCACT | CATCACGCCCACGAGAGAAA |
| MYL5 | CTGTTTGGGGAGAAGCTGA | AGGCGAACTGGAACATCTG |
| PPP1R12C | GGACTCACTGACCCATGCG | AGCTTCTTTTTGGTTCCGAAGG |
| RENBP | AAGGTGCAGCGAACCATCTTC | AGTGGACGATCTGATCCATCAT |
| TP53I13 | TAGGGAGAGTAATGCCCCATC | GCCTGACTTGAACAGACACAG |
| CPB1 | GGCACTCTTGGTTCTGGT | GTCAATCTGGGTCGTGCT |
| GTF2I | TTGTCGTCGGAACTGAAAGAG | CGATTTGCCTGGGTTGTAGAT |
| HAUS7 | AGCTCACTGAAAGGGGTCC | ATCGAGCAACTGGTCCATGAA |
| IDUA | GTCAGCAGGAAGCCATCG | GGCACAGGGTCCGAGAA |
| IL6 | ACTCACCTCTTCAGAACGAATTG | CCATCTTTGGAAGGTTCAGGTTG |
| PLAC9 | GAGCACAGCGTGTGACAGA | GATCCACGGTCTTCTCTACCA |
| TAZ | CCCTTTCTAACCTGGCTGT | CTTGCTCTGCTCCATCACT |
